# Sevoflurane exposure in early life: mitochondrial dysfunction and neurotoxicity in immature rat brains without long-term memory loss

**DOI:** 10.1038/s41598-024-79150-3

**Published:** 2024-11-20

**Authors:** Lin Qiu, Hongwei Li, Bingbing Li, Joakim Ek, Xiaoli Zhang, Yiwen Chen, Zehua Shao, Jie Zhang, Jiaqiang Zhang, Hongqi Lin, Changlian Zhu, Yiran Xu, Xiaoyang Wang

**Affiliations:** 1 Department of Anesthesia, Henan Provincial People’s Hospital, Department of Anesthesia of Central China Fuwai Hospital, Central China Fu Wai Hospital of Zhengzhou University, Zhengzhou, 450003 Henan China; 2 Zhengzhou University People’s Hospital, Henan Provincial People’s Hospital, Zhengzhou, 450003 China; 3 Henan Key Laboratory of Child Brain Injury and Henan Pediatric Clinical Research Center, Institute of Neuroscience, Third Affiliated Hospital of Zhengzhou University, Zhengzhou, 450052 China; 4 Department of Laboratory Medicine, Third Affiliated Hospital of Zhengzhou University, Zhengzhou, 450052 China; 5 Zhengzhou Key Laboratory for In Vitro Diagnosis of Hypertensive Disorders of Pregnancy, Zhengzhou, 450052 China; 6 Perinatal Center, Department of Physiology, Institute of Neuroscience and Physiology, University of Gothenburg, Gothenburg, 40530 Sweden; 7 Department of Surgery, Fuwai Hospital, National Center for Cardiovascular Diseases, Chinese Academy of Medical Sciences and Peking Union Medical College, Beijing, 100037 China; 8 Center for Brain Repair and Rehabilitation, Institute of Neuroscience and Physiology, University of Gothenburg, Gothenburg, 40530 Sweden; 9 Centre of Perinatal Medicine & Health, Department of Obstetrics and Gynaecology, Institute of Clinical Sciences, Sahlgrenska Academy, University of Gothenburg, Gothenburg, 41685 Sweden

**Keywords:** Sevoflurane, Metabolomics, Transcriptomics, Immature brain, Neurotoxicity, Biochemistry, Molecular biology, Neuroscience, Physiology, Medical research, Molecular medicine, Neurology

## Abstract

Neurotoxic effects of general anesthetics, particularly sevoflurane, on pediatric neurodevelopment are a global concern. This study investigated the molecular and metabolic impacts of repeated short exposures to sevoflurane in neonatal rats. Metabolomics analysis revealed significant changes in fatty acid and mitochondrial energy metabolism. Transcriptomic analysis identified altered gene expression related to neurodevelopment and mitochondrial function. Various analyses emphasized upregulation in oxidative phosphorylation and DNA repair pathways. Weighted gene co-expression network analysis (WGCNA) identified key gene modules associated with sevoflurane exposure. Despite these acute changes, no significant long-term memory impairments were detected. These findings highlight the impact of sevoflurane on mitochondrial energy metabolism, oxidative stress, and neuroinflammation, emphasizing its relevance to pediatric neurodevelopment. The absence of substantial long-term memory impairments provides insights into the safety and implications of sevoflurane use in pediatric anesthesia, calling for further research.

**Supplementary Information:**

The online version contains supplementary material available at 10.1038/s41598-024-79150-3.

## Introduction

The postnatal development of the central nervous system is a period of dynamic and essential growth involving neural stem cell proliferation, synapse formation, and the establishment of neural networks. This developmental stage is particularly susceptible to environmental influences. Annually, a notable volume of surgical procedures is carried out on children younger than three, which accentuates the critical need for more refined anesthetic practices. The neurotoxic potential of anesthetics like sevoflurane in early childhood is a significant concern, with associations between neonatal surgical anesthesia and subsequent developmental issues^1–5^. Despite the variability in surgical procedures and the necessity for post-operative sedation, the use of anesthetics is common. In response to emerging evidence, the US Food and Drug Administration (FDA)’s safety warning highlights the need for careful consideration of anesthetic exposure in pediatric neurology^6^, particularly regarding the effects of prolonged or repeated use on cognitive development. This backdrop calls for a thorough investigation into the neurological effects of anesthetics, considering the sensitivity of developing brains to such external factors.

Sevoflurane, known for its mild odor and rapid clearance, has become a preferred anesthetic in pediatric care, especially for neonates and infants with developing organ systems. While its pharmacokinetics suggest a safer profile with minimal residual accumulation, the implications of sevoflurane on neurodevelopment are increasingly examined^7^. Studies indicate that anesthetic exposure during pivotal brain growth stages could induce a range of neurotoxic effects, from cellular and structural brain alterations to disruptions in neurotransmitter signaling and ion channel function^8–12^. These effects include alterations in microglial function, compromise of the blood-brain barrier (BBB), changes in gut microbiota composition, and modulation of cholinergic neurotransmission^13^. These findings have fueled concerns over long-term cognitive outcomes^14^. Moreover, the regulatory mechanisms underlying these effects are not well understood, presenting a substantial challenge in comprehensively understanding sevoflurane’s pharmacological profile.

Our current investigation utilizes a validated neonatal rat model of anesthesia^11,12^ to conduct a thorough metabolomics assessment coupled with comprehensive RNA sequencing of the immature rat brain. This approach is aimed at unveiling the potential neurologically impactful molecular and cellular metabolic mechanisms attributable to sevoflurane exposure.

## Materials and methods

### Anesthesia process by sevoflurane

The pregnant Sprague-Dawley (SD) rats were housed at Experimental Animal Center of Zhengzhou University and maintained in an environment with controlled temperature and humidity and subjected to a 12-hour light-dark cycle. They had ad libitum access to water and food. For the study, a total of 80 rat pups, evenly split between males and females, were used at postnatal day 7 (PND7). Of these, 40 were designated for short-term metabolome and transcriptome analysis, with 20 rats allocated to each. For both analyses, there were 10 rats in the sevoflurane-treated group and 10 in the control group, with an equal distribution of males and females in each group. An additional 40 rats were used for long-term behavioral assessments, with 20 sevoflurane-treated and 20 control rats, again maintaining an equal number of males and females in each group (Supplementary Fig. 1). Animal experiments conformed to the Guidelines on Welfare and Ethical Review for Laboratory Animals established by the Standardisation Administration of the People’s Republic of China (2018). This study received approval from the Zhengzhou University Ethics Committee for Laboratory Animal Welfare and Management (ethical number 2020025).

Accordance: We confirmed that all experiments in this study were performed in accordance with the relevant guidelines and regulations.

Arrive: All the procedure of the study is followed by the ARRIVE (Animal Research: Reporting In Vivo Experiments) guidelines.

**Fig. 1:**
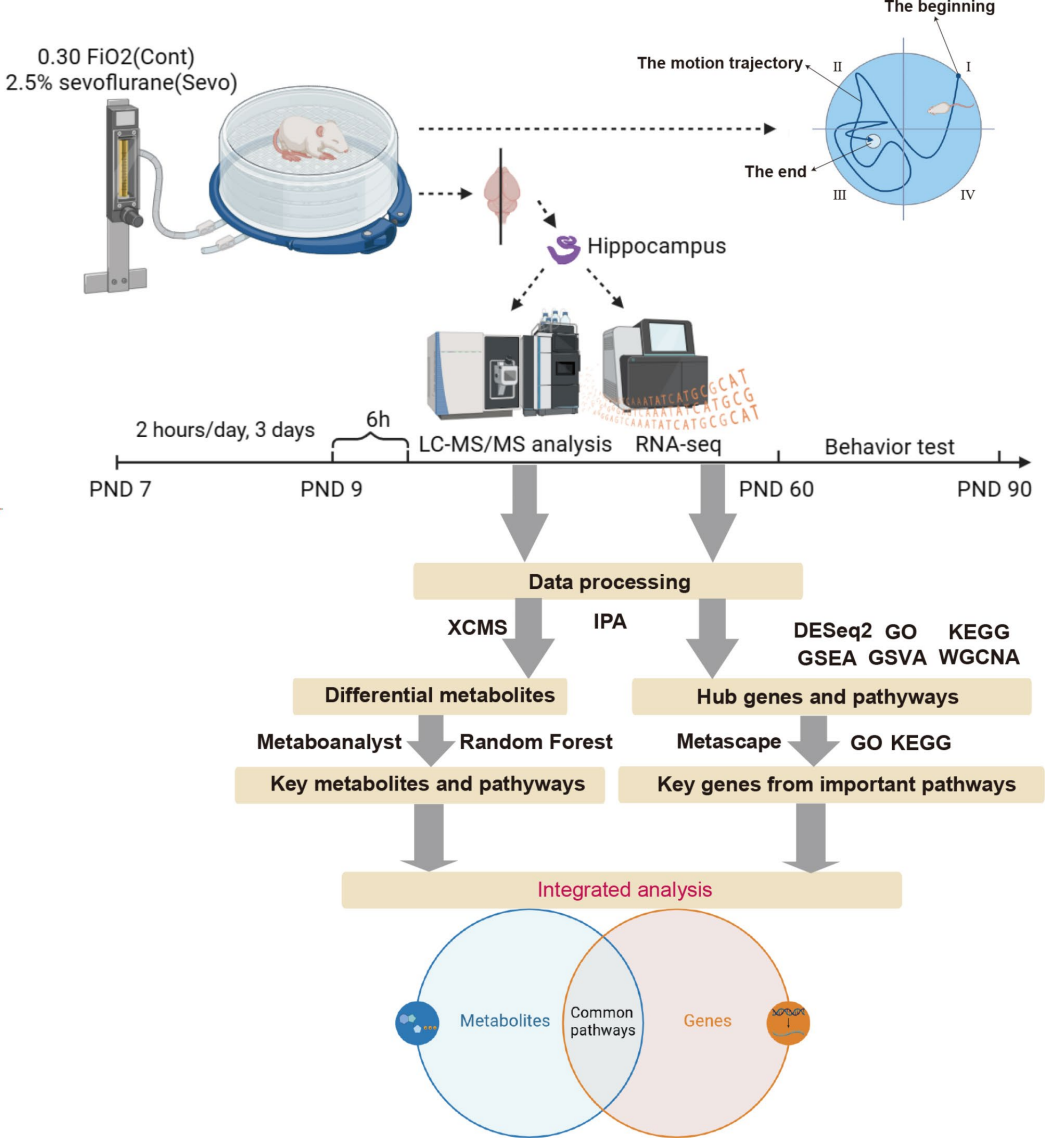
The diagram illustrating the flow of the study.

PND7 rat pups were randomly allocated into control and sevoflurane anesthesia groups. Randomization was implemented within litters using a computer-generated random number table, assigning pups to either the experimental or control group to balance representation across different litters. Blinding was maintained throughout data collection and analysis, with researchers unaware of group assignments until after the completion of data analysis to minimize subjective bias. Pups were exposed to for 2 h daily for 3 days in a chamber to either 0.30 FiO_2_(control) or 2.5% sevoflurane (Shanghai Hengrui Medicine Co., LTD) in 0.30 FiO_2_ with a 4 L/min flow rate^11^. A total of 80 animals were exposed in batches, with a group of pups placed in the chamber at each time. Chamber concentrations of CO_2_, oxygen, and sevoflurane were monitored. Arterial pulse oximetry was continuously recorded from one randomly selected animal in each batch. Monitoring across different batches ensured consistent oxygen level control and confirmed that hypoxia was not a confounding factor during sevoflurane exposure. Pups were checked every 5 min. Body temperature, maintained using a homeothermic pad, was confirmed with a rectal probe to be around 36.5℃. Post sevoflurane anesthesia, pups were warmed until their righting reflex returned. They were then returned to their home cage with the dam until the time of sacrifice. On PND 9, 6 h after the final sevoflurane anesthesia session, the animals were sacrificed by an overdose of pentobarbital (100 mg/kg intraperitoneally) and perfused transcardially with cold saline. The brains were harvested, the hippocampus was isolated, and the tissue was prepared for subsequent metabolic analysis and total RNA sequencing (Fig. 1).

### Sample preparation for metabolomics analysis

Twenty rat pups, equally divided by gender, were assigned to experimental and control groups. Metabolites were extracted from isolated rat hippocampal samples obtained on PND 9. Samples were flash-frozen in liquid nitrogen for 15 min and stored at -80℃ until analysis. Each 25 mg sample was mixed with a 500 µL extraction solution (acetonitrile: methanol: water, 2:2:1) containing an isotopically-labelled internal standard mixture. After vortexing for 30 s, samples underwent homogenization at 35 Hz for 4 min, followed by sonication in an ice-water bath for 10 min. This cycle was repeated thrice. A 200 µL supernatant aliquot was used for analysis after homogenization, incubation, and centrifugation. Quality control was ensured by pooling 10 µL of each sample to create a composite quality control (QC) sample. Liquid chromatography-tandem mass spectrometry (LC-MS/MS) analysis employed an ultra-high-performance liquid chromatography (UHPLC) system with a UPLC BEH Amide column connected to a Q Exactive HF-X mass spectrometer. The mobile phase consisted of ammonium acetate, ammonia hydroxide, and acetonitrile. The Q Exactive HF-X mass spectrometer operated in information-dependent acquisition mode, continuously evaluating the full scan MS spectrum. Source conditions included specific parameters for gas flow rate, temperature, resolution, collision energy, and spray voltage.

### Metabolomics data analysis

The raw data was converted to mzXML format using ProteoWizard and processed with an in-house program developed in R, based on XCMS. This program facilitated peak detection, extraction, alignment, and integration. Normalized metabolite concentrations underwent cluster analysis for pattern identification, principal component analysis (PCA) for data structure visualization, and orthogonal partial least squares-discriminant analysis (OPLS-DA) to identify discriminative metabolites. Overfitting was evaluated through 200 permutation tests^15^ to determine the OPLS-DA model’s fit degree. T-tests were used alongside OPLS-DA to screen potential biomarkers associated with sevoflurane exposure. Significantly different metabolites between groups were identified based on *p* < 0.05 and variable importance in projection (VIP) > 1 criteria. The VIP scores were calculated as part of the standard OPLS-DA analysis in MetaboAnalyst 5.0, where the score quantifies the contribution of each metabolite to group separation. A VIP score greater than 1 indicates a significant contribution to the model, ensuring the robust identification of key metabolites. These stringent criteria were implemented to avoid overfitting or false-positive errors, thus enhancing the robustness and reliability of the results. Metabolite annotation was performed using the in-house MS2 database (BiotreeDB) with a cutoff set at 0.3. Enrichment analysis of metabolic pathways for differential metabolites was conducted using MetaboAnalyst 5.0 (https://www.metaboanalyst.ca/) based on the Kyoto Encyclopedia of Genes and Genomes (KEGG) pathway database. In line with the exploratory nature of this study, uncorrected *p*-values were used for pathway analysis to capture a broader range of altered pathways. Metabolomics data was detected in both positive and negative ionization modes, and the combined data from both modes were used for analysis.

### RNA isolation and sequencing

Twenty rat pups were divided into experimental and control groups, with an equal distribution of males and females. Total RNA was extracted from the PND9 rat hippocampus, and sequencing libraries were prepared using the NEBNext^®^ UltraTM RNA Library Prep Kit for Illumina^®^. mRNA isolation, cDNA synthesis, adapter ligation, and size selection were performed to obtain fragments of 250–300 bp. Cluster generation and sequencing were conducted on an Illumina Novaseq platform, resulting in 150 bp paired-end reads.

### RNAseq data analysis

In the transcriptomic analysis, we used the DESeq2 R package, which provides an integrated approach for controlling multiple comparisons. The Benjamini-Hochberg False Discovery Rate (FDR) method was employed to adjust *p*-values, minimizing the risk of Type I errors. This statistical adjustment ensures that only genes with corrected p-values below the defined threshold (FDR-adjusted *p*-values < 0.05) were considered differentially expressed. This rigorous correction method reduces the likelihood of false positives, allowing for the accurate identification of differentially expressed genes (DEGs). The final list of significant genes was based on these adjusted *p*-values, ensuring robust and reliable transcriptomic results in the context of our study.

The biological functions and corresponding pathways of the identified DEGs were assessed using Gene Ontology (GO), KEGG^16^, Gene Set Enrichment Analysis (GSEA)^17^ and Gene Set Variation Analysis (GSVA)^18^ at a significance level of *p*_*adjusted*_ < 0.05. The online database Metascape (http://metascape.org/gp/index.html#/main/step1) and DAVID (https://david.ncifcrf.gov/home.jsp) were also employed to predict gene function and pathway. The KEGG, GSEA and GSVA were performed in R software, utilizing the R packages “clusterProfiler” (version 4.2.2)^19^ and “msigdbr” (version 7.5.1). The obtained results were visualized using the R packages “enrichplot” (version 1.14.2) and “ggplot2” (version 3.3.6).

we constructed a co-expression network using the WGCNA (version 1.72.1) package^20^ in R software. To emphasize strong correlations and achieve a standard scale-free network, we utilized the pickSoftThreshold function to calculate an appropriate soft threshold parameter, β. This was determined through scale independence and mean connectivity analysis of modules, yielding a β value within the range of 1 to 20. Hierarchical clustering was then performed, resulting in the identification of co-expression modules and the generation of a hierarchical clustering tree. We set a minimum module size of 30 genes and proceeded to merge modules with a correlation greater than 80%. These steps allowed us to construct a comprehensive co-expression network, enabling the identification of gene modules and their relationships.

### Ingenuity pathway analysis

The significant DEGs generated from total RNAseq, as well as metabolites generated from the metabolic analysis (cut-off adjusted *p* value of < 0.05 for metabolites and *p* < 0.001 for DEGs was applied in order to have an appropriate number of molecules as input), were further analyzed using Ingenuity Pathway Analysis (IPA, Qiagen; Version 101138820).

The powerful “core analysis” function was then employed to gain comprehensive insights from this differential expression data. Through this analysis, we were able to explore various aspects, including downstream biological functions, canonical pathways, upstream transcriptional regulators, and networks associated with the regulated molecules. It is worth noting that each identifier was accurately matched to its respective object within the extensive Ingenuity Pathway Knowledge Base (IPKB).

### Integrated transcriptomic and metabolomic analysis

KEGG co-enrichment analysis and Joint pathway analysis was used to integrate the metabolite and gene data in response to sevoflurane exposure. We simultaneously mapped the differentially expressed genes and metabolites of the two groups to the KEGG pathway to better understand their biological relationships. At the same time, we sorted and plotted differentially expressed genes and metabolites by *p* value, focusing on pathways with *p* values < 0.05 for both gene and metabolite pathways. Joint pathway analysis was conducted using MetaboAnalyst 5.0.

### Morris water maze tests

We conducted two sets of tests, one during PND 60–63 and the other at PND 87–90, with *N* = 20 per group, meaning 20 rats in the sevoflurane exposure group and 20 in the control group, equally distributed by sex. Rats were identified by ear notching and were dried after each training session before being placed in a new breeding box. The experimenter recorded each rat’s identity, ear notch, and cage number in a notebook for reference. In the morris water maze assessment, the apparatus was filled with water made opaque using titanium dioxide, with a water level maintained 1.0 cm above a 10 cm diameter platform. The water temperature was consistently kept at 22 °C in a quiet environment. The round swimming pool was divided equally into four quadrants (I, II, III, and IV), with the target platform located in quadrant III (Fig. 1). The platform remained in the same position throughout the training days and was removed on the testing day. The pool had a diameter of 150 cm, the target platform was positioned 32 cm below the water surface, and the water depth was 33 cm. The rats were released from the middle part of the marginal area of quadrants I, II, or IV during the training days and were only released from quadrant II on the testing day. For spatial learning evaluation, the rats underwent training from PND 60–63, and PND 87–90, involving three daily trials to locate the platform, with escape latency times recorded. On PND 63 and PND 90, during the memory function assessment, the platform was removed. We measured the mean distance to the original platform location, the number of times the platform area was crossed, and the duration spent in the fourth quadrant (where the platform was located). The repeated measures two-way ANOVA was employed for statistical analysis.

## Results

Metabolomic analysis demonstrates metabolic alterations and neurotoxic effects of sevoflurane in immature rat brains.

Metabolic analysis showed that, six hours after the final exposure to sevoflurane, 5,919 metabolite features were identified in the positive ionization mode and 6,672 in the negative ionization mode. The metabolomic dataset underwent quality and analytical performance evaluation using PCA and OPLS-DA (Supplementary Fig. 2A-D). In positive ionization mode (Supplementary Fig. 2A, C), both PCA score plots and OPLS-DA models showed clear separation between sevoflurane and control groups. The OPLS-DA model exhibited distinct group differences, validated by 200 permutation tests ensuring reliable R^2^Y values and strong predictability (Q^2^), ruling out overfitting (Q^2^ < 0). Likewise, in negative ionization mode, effective discrimination between the two groups was observed in both PCA and OPLS-DA models (Supplementary Fig. 2B, D).

In the positive ionization mode (Supplementary data 1), there were 213 metabolites identified with increased levels and 378 with decreased levels in the sevoflurane-treated group. Similarly, in the negative ionization mode (Supplementary data 2), the sevoflurane-treatment group exhibited 806 upregulated and 98 downregulated metabolites in comparison to the control group. The heatmap illustrates the alterations in metabolic profiles of rat hippocampal tissue following sevoflurane exposure, compared to control samples, in the positive or negative ionization mode (Supplementary Fig. 2E, F).

**Fig. 2:**
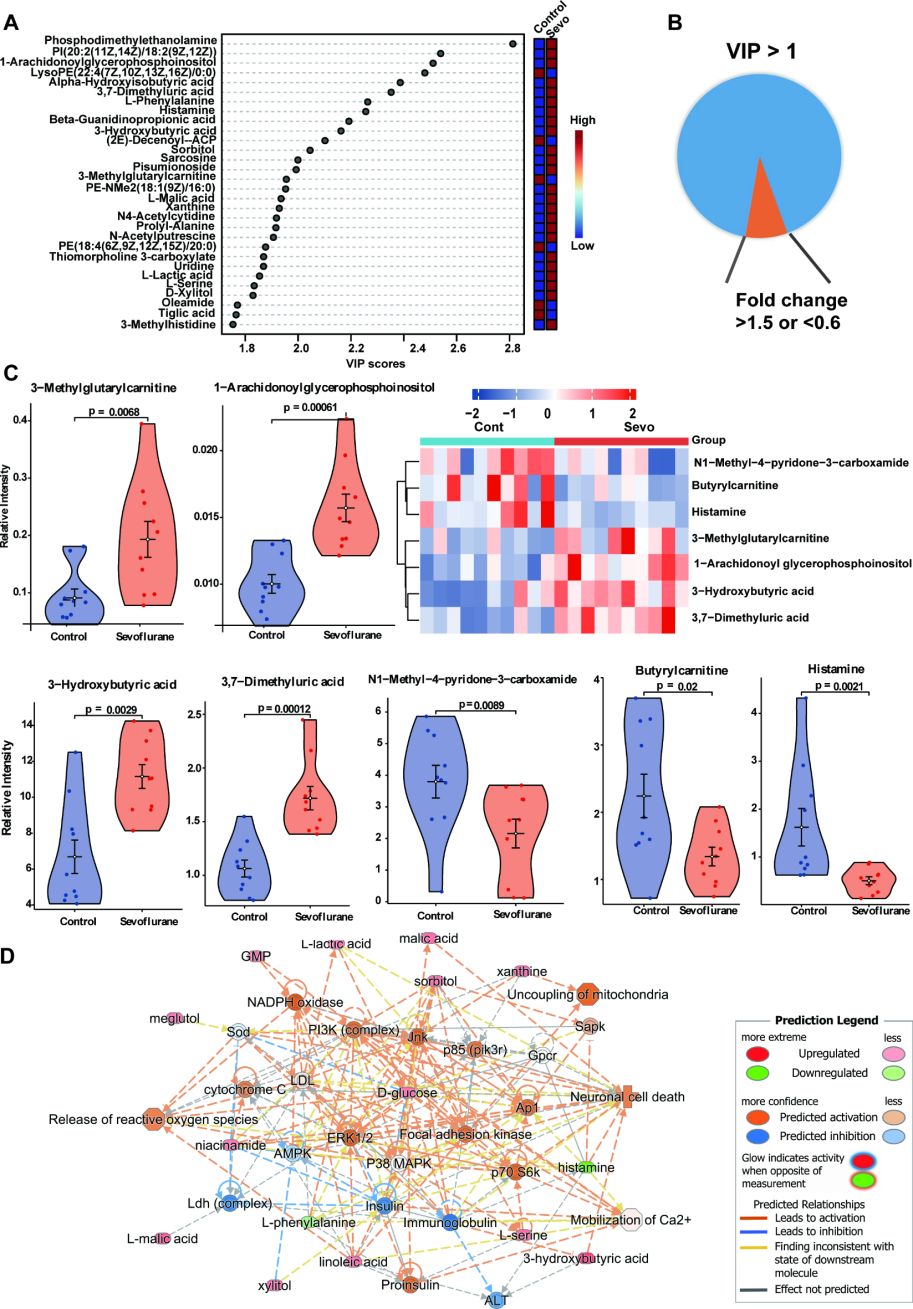
Altered metabolites associated with mitochondrial dysfunction in sevoflurane-induced neurotoxicity. (**A**) Top 30 metabolites with VIP score > 1 identified using OPLS-DA analysis. These metabolites were the most influential in separating the sevoflurane-treated group from controls. (**B**, **C**) Heatmap (**B**) and violin plot (**C**) showing significant metabolic variations between sevoflurane-treated and control groups. Metabolites with *p* < 0.05, VIP > 1, and fold change thresholds (> 1.5 or < 0.6) are highlighted. The heatmap represents relative abundances across the sample groups, while the violin plot illustrates the distributions of key metabolites between groups. **D** IPA network analysis integrating the key metabolites identified in parts A-C with transcriptomic data. The network highlights interactions between these metabolites, genes, and relevant pathways involved in mitochondrial function and neurotoxicity. Nodes represent metabolites or genes, while edges indicate known interactions. The color coding (orange for predicted activation, blue for predicted inhibition) indicates the predicted effects of sevoflurane exposure on these pathways.

In the subsequent analysis, we utilized T-test for univariate data analysis andVIP values from the OPLS-DA models to identify the most significantly altered metabolites. There were 77 metabolites (VIP > 1, and FDR-adjusted *p*-value < 0.05) different significantly between the groups (Supplementary Table S1) (Supplementary data 3). The top 30, ranked by VIP values, are presented in Fig. 2A, representing various categories including organic acids, lipids, organoheterocyclic compounds, organic nitrogen and oxygen compounds, and nucleosides.

To refine these differential metabolites, we applied an additional criterion require a fold change greater than 1.5 or less than 0.6 (Fig. 2B). This approach identified 7 metabolites as the most significantly altered, as depicted in Fig. 2C. Notably, levels of 3-methylglutarylcarnitine, 3-hydroxybutyric acid, 3,7-dimethyluric acid, and 1-arachidonoylglycerophosphoinositol were significantly increased (*p* < 0.05, fold change (FC) > 1.5), while butyrylcarnitine, N1-methyl-4-pyridone-3-carboxamide, and histamine in hippocampal tissue were reduced (*p* < 0.05, FC < 0.6) following sevoflurane exposure.

Supplementary Table S2 outlines key metabolites, such as 3-methylglutarylcarnitine and butyrylcarnitine, integral to fatty acid metabolism, impacting mitochondrial energy metabolism and the tricarboxylic acid (TCA) cycle. These metabolites are linked with phenomena like brain injury and emergence agitation following anesthesia. 3-hydroxybutyric acid, a key ketone in lipid metabolism, relates to psychiatric disorders, epilepsy, and sleep disturbances. 3,7-dimethyluric acid, involved in caffeine metabolism, influences cognitive function. N1-methyl-4-pyridone-3-carboxamide, vital for mitochondrial oxidative pathways, highlighted. Moreover, histamine and 3-hydroxybutyric acid are connected to neuroinflammation, with histamine dysregulation observed in neurodegenerative diseases.

KEGG analysis revealed concentrated metabolic alterations following sevoflurane exposure, particularly in pathways including caffeine metabolism, aminoacyl-tRNA biosynthesis, fructose and mannose metabolism, galactose metabolism, as well as glycine, serine, and threonine metabolism. Changes were also observed in ketone bodies synthesis and degration, butanoate metabolism, phenylalanine, tyrosine, and tryptophan biosynthesis, phenylalanine metabolism, pyruvate metabolism, and TCA cycle, as shown in Supplementary Fig. 3 and supplementary data 4. Notably, these pathways are implicated in mitochondrial energy metabolism and neurotoxicity.

Our in-depth analysis utilizing QIAGEN IPA focused on causal networks, emphasizing the “Top Network” (Fig. 2D). This network predicted downstream effects intricately linked with mitochondrial energy metabolism and neuronal outcomes, including neuronal death and excitation. Key processes identified included ROS release, mitochondria uncoupling, Ca^2+^ mobilization, NADPH oxidase activity, and ERK 1/2 activation. Canonical pathways from IPA’s supplementary data supported these findings, highlighting lactate fermentation from pyruvate, the eukaryotic TCA cycle, and glycine/serine biosynthesis as crucial for cellular energy production, protein assembly, metabolic regulation, and mitochondrial energy production (Supplementary data 5). Our IPA analysis pinpointed upstream regulators such as GNMT and IL-37, suggesting their pivotal role in sevoflurane’s metabolic and inflammatory. Their z-scores indicate a complex interaction where sevoflurane modulates both metabolic functions and inflammatory pathways, providing insight into its dual effect on neurodevelopment and neurotoxicity.

Altogether, our extensive metabolomic analyses demonstrate that repeated sevoflurane exposure induces significant alterations in key metabolic pathways, particularly impacting mitochondrial energy metabolism, oxidative stress, and neuroinflammation. These changes are intricately linked to neurodevelopmental abnormalities and neurotoxic effects.

RNA Sequencing unveils sevoflurane’s effects on mitochondrial dysfunction, neurodevelopmental impairment, and neurotoxicity in immature rat brains.

To understand how gene expression changes affect metabolite variations, transcriptomics is crucial for unraveling the mechanisms behind observed metabolomic shifts. Hence, we conducted an in-depth transcriptome analysis of the rat brain hippocampus six hours after the last sevoflurane exposure.

We initiated a differential gene expression analysis using DESeq2 to compare sevoflurane and control groups (Supplementary data 6). The PCA indicated clear data separation and robustness (Supplementary Fig. 4A). A total of 2264 genes showed increased expression and 2592 showed decreased expression (Supplementary Fig. 4B). The top 18 DEGs highlighted in heatmap (Supplementary Fig. 4C), included six genes crucial for neurodevelopment: *Pax4*, *Gata3*, *Lgals5*, *Mobp*, *Lingo4*, and *Aldh18a1*. These genes play distinct roles in neuronal development and functionality, ranging from *Mobp*’s involvement in structural myelin components to the wider regulatory functions in cellular differentiation and survival exhibited by the others.

To explore the potential impacts of sevoflurane exposure on biological processes and signaling pathways, we conducted in-depth analyses, including GO, KEGG, GSEA, and GSVA. These analyses were based on the DEGs.

**Fig. 3:**
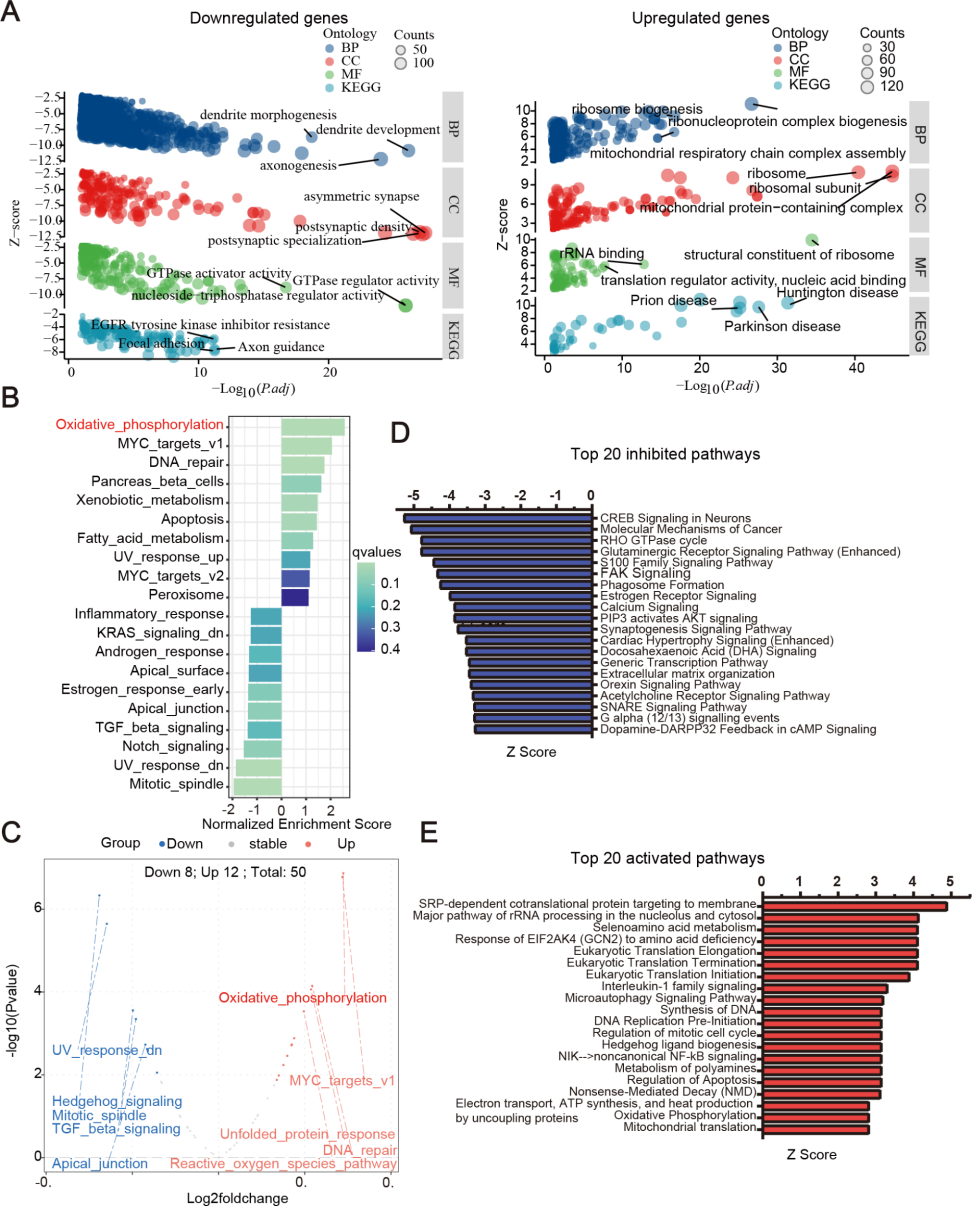
RNA sequencing identified sevoflurane induced alterations linked to mitochondrial function, neuronal development and neurotoxicity. (**A**) GO and KEGG, *p*. adj < 0.05; (**B**) GSEA, (**C**) GSVA, and (**D**, **E**) Top 20 canonical pathways analysis by IPA: (**D**): inhibited pathways (z score ≤ -2); (**E**): activated pathways (z score ≥ 2).

In the GO and KEGG analyses (Fig. 3A, Supplementary data 7), we identified DEGs primarily involved in neuronal morphogenesis, including axonogenesis, dendrite development, and dendrite morphogenesis. These genes played a role in synaptic specialization particularly in post-synaptic aspects, and were associated with various neurological disorders. Furthermore, these genes were linked to mitochondrial functionality, emphasizing mitochondrial respiratory chain complex assembly and mitochondrial protein complexes, as well as RNA metabolism, which covers aspects like translation regulator activity, nucleic acid binding, and ribosome biogenesis. Cellular signaling regulation, particularly GTPase regulator and activator activities, was also a significant focus.

GSEA analysis (Supplementary Fig. 4B) highlighted notable upregulation in pathways including oxidative Phosphorylation, MYC Targets V1, DNA Repair, and apoptosis. While pathways like the mitotic spindle and UV response DN showed clear downregulation. GSVA analysis (Supplementary Fig. 4C) identified 12 significantly upregulated pathways, notably oxidative Phosphorylation, DNA repair, and ROS pathways, and 8 downregulated pathways, including UV response DN, mitotic spindle, and TGF beta signaling. Both analyses consistently showed an upregulation in oxidative phosphorylation and DNA repair pathways and a downregulation in the mitotic spindle and UV response DN pathways. Notably, oxidative phosphorylation was the most significantly enriched pathway, underscoring a strong link to mitochondrial dysfunction (Supplementary data 8).

In our IPA analysis of DEGs, we identified pathways related to mitochondrial function and neuronal development (Supplementary data 9). These included pathways with inhibition (Supplementary Fig. 4D) (e.g., Glutaminergic Receptor Signaling) and activation (Supplementary Fig. 4E) (e.g., CREB Signaling in Neurons) affecting processes like oxidative phosphorylation, electron transport, and synaptogenesis. Additionally, activated pathways in RNA metabolism and DNA repair, such as Eukaryotic Translation Termination and DNA Replication Pre-Initiation, were observed. The analysis also highlighted disruptions in neuronal development and functioning, linking to neurodegenerative diseases, congenital malformations, and cognitive impairments. Additionally, it is worth noting that the top biological processes and pathways affected were similar in both sexes (Supplementary Fig. 5). Moreover, learning and cognition were identified as significantly affected, with potential implications for motor and cognitive disorders (Supplementary Fig. 6, Supplementary data 10). Upstream regulator analysis suggested several activated (MIR17HG, CSF3, NFE2L1) and inhibited (CNR1, ERG, PAX2, PTF1A, CDK6) regulators as potential drivers of these changes Supplementary Fig. 7, Supplementary data 11), indicating directions for future research.

Altogether, our in-depth analysis, encompassing GO, KEGG, GSEA, GSVA, and IPA, reaffirms that sevoflurane exposure significantly alters gene expression related to neuronal development, mitochondrial function, and various neurological disorders. Key findings include the upregulation of oxidative phosphorylation and DNA repair pathways, and notable associations with neuronal apoptosis and cognitive impairments. These findings were validated using a different set of key genes, generating a heatmap for confirmation (Supplementary Fig. 8). These findings collectively suggest potential neurotoxicity, emphasizing critical areas for future research and underscoring the importance of understanding sevoflurane’s effects on the nervous system.

**Fig. 4:**
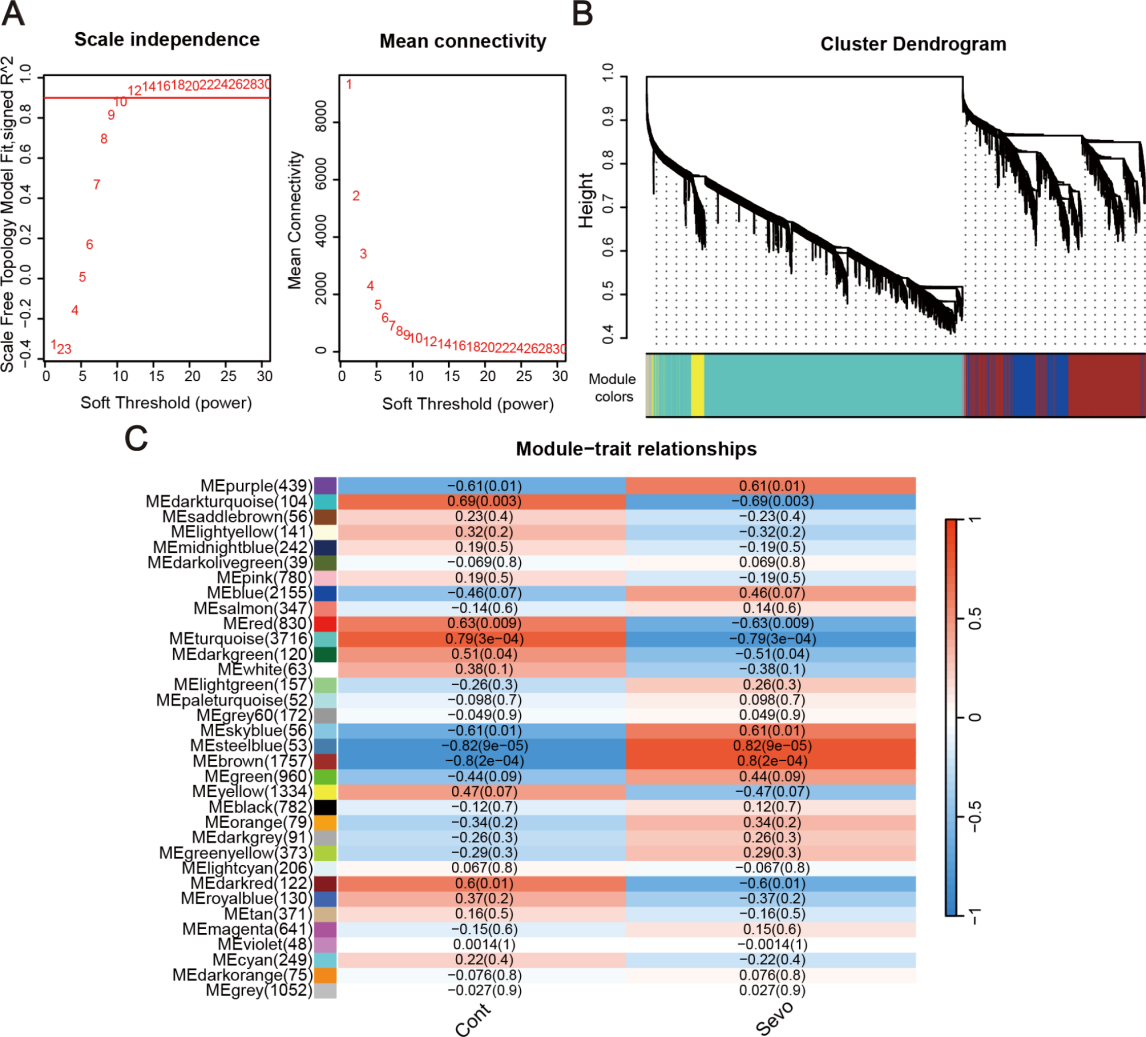
Identification of sevoflurane exposure-related modules in WGCNA. (**A**) Gene-to-gene correlation calculations for soft threshold (β) and scale-free fit assessment (R-squared). (**B**) Cluster dendrogram construction identifying network modules. (**C**) Heatmap of module eigengenes correlation with different stages.

Using the R package WGCNA for gene modules identification, we aimed to reveal the molecular mechanisms behind sevoflurane’s neurotoxicity by grouping genes based on co-expression patterns. Our analysis established a scale-free network with β = 12, achieving a scale independence of 0.9 and reduced mean connectivity (Fig. 4A). Genes were hierarchically clustered into modules, with a clipping height threshold at 0.25, identifying key modules like yellow, brown, turquoise, and blue (Fig. 4B). Strong correlations between these modules and groups (|r| > 0.8), especially the steelblue (*r* = 0.82, *p* = 9E-05) and brown modules (*r* = 0.80, *p* = 2E-04), indicated their significant roles in response to sevoflurane (Fig. 4C).

Hub genes, highly connected within networks, play a vital role in biological processes. Their identification is key to understanding cellular responses to sevoflurane exposure. We identified hub genes related to sevoflurane exposure by intersecting DEGs with genes from the steelblue and brown modules, visualized using Venn diagrams. This revealed 20 (Supplementary Fig. 9A) and 1133 (Supplementary Fig. 9B) hub genes, respectively. GO and KEGG analysis of the 20 hub genes highlighted their role in DNA repair, including activities like DNA- (apurinic or apyrimidinic site) endonuclease activity and base excision repair. Analysis of the 1133 genes using Metascape underscored sevoflurane’s impact on RNA metabolism (including ribonucleoprotein complex biogenesis) and mitochondrial energy metabolism (involving the TCA cycle and mitochondrial electron transport).

Integrated transcriptomic and metabolomic analysis reveals sevoflurane-induced pathway alterations in pyruvate metabolism and amino acid biosynthesis.

To thoroughly understand sevoflurane’s effects and unravel the complex interplay between gene expression changes and metabolic pathways, we conducted an integrated analysis combining RNAseq and metabolic data. KEGG co-enrichment analysis identified sevoflurane-induced impacts on various biological pathways at both gene expression and metabolic level. Significant pathways affected by sevoflurane exposure included caffeine metabolism, aminoacyl-tRNA biosynthesis, and various metabolic processes involving fructose, mannose, galactose, glycine, serine, threonine, tryptophan, pyruvate, and the citrate cycle (Supplementary data 12). Despite the absence of significantly co-enriched pathways with *p*-values < 0.05 in the positive mode, pathways such as aminoacyl-tRNA biosynthesis, valine, leucine, and isoleucine metabolism, lysine degradation, and beta-alanine metabolism were noted for their relatively lower *p*-values, nearing *p* < 0.05, indicating potential alterations due to sevoflurane exposure.

**Fig. 5:**
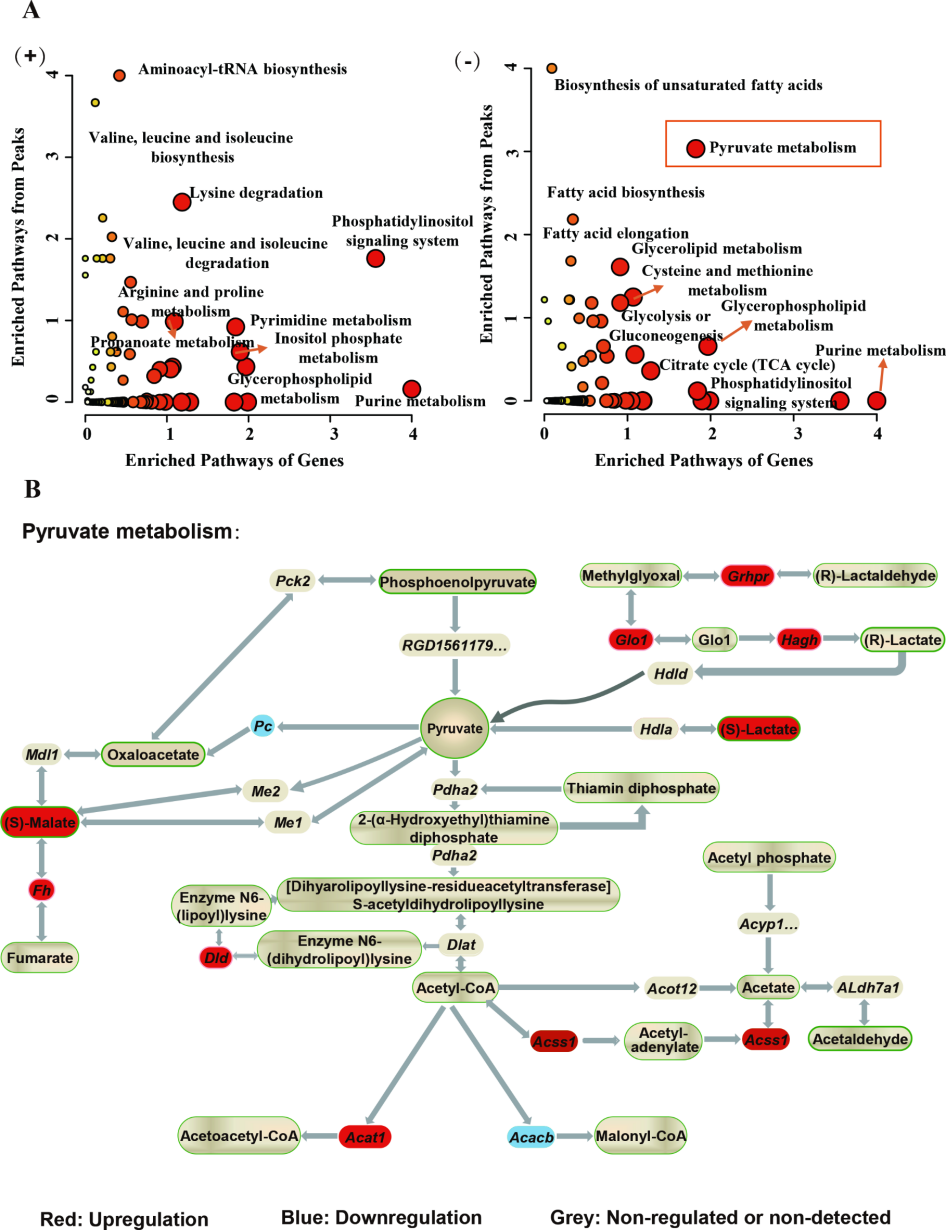
Integrated RNAseq and metabolomic analysis: joint pathway enrichment analysis on significantly dysregulated metabolites and genes. (**A**) The overlapping metabolites-genes pathways determined by integrated pathway enrichment analysis in MetaboAnalyst. Symbols ‘(+)’ and ‘(−)’ represent positive and negative ionization modes, respectively. (**B**) Mapping of dysregulated metabolites and genes in “Pyruvate Metabolism”; red for up-regulation, blue for down-regulation, gray for non-dysregulated or non-detected.

In the negative ionization mode, a notable change in pyruvate metabolism (*p* < 0.05) was observed (Supplementary Fig. 10). Joint pathway enrichment consistently linked gene and metabolite data to pyruvate metabolism, as depicted in Fig. 5A and B. Details on the genes and metabolites linked to these differentially enriched pathways are provided in Supplementary Table S3. Moreover, we constructed a network that closely links the important metabolic products of pyruvate metabolism, L-lactic acid, and L-malic acid, with DEGs and disease prediction. As depicted in Supplementary Fig. 11, these two dysregulated metabolites pointed towards neurologic and mitochondrial disorders, with their disease associations also confirmed by the online DAVID database (Supplementary data 13). In summary, the integrated approach highlights significant impacts on pyruvate metabolism and amino acid biosynthesis, offering crucial insights into specific molecular mechanisms and pathways.

Early life sevoflurane exposure does not cause long-term neurobehavioral changes.

**Fig. 6:**
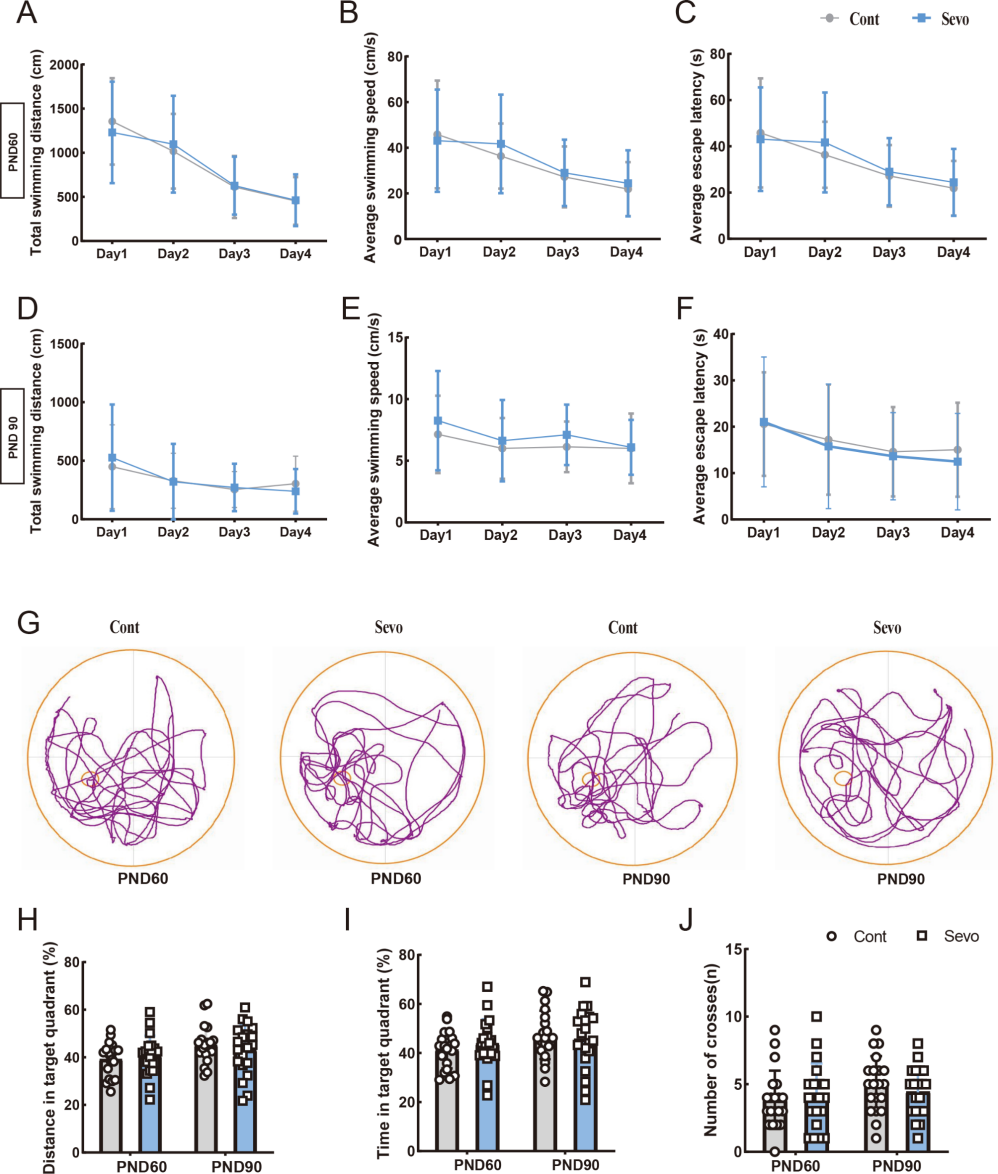
The learning and memory ability in sevoflurane-induced rats using morris water maze test, which includes 20 samples for the sevoflurane exposure group and 20 samples for control group. The tests conducted at two time points: the first at PND60 (1st time point) and the second at PND90 (2nd time point). Two-way repeated measures ANOVA was used for statistical analysis. (**A**–**C**) The total swimming distance, average swimming speed, and average escape latency of the first four training days at PND60. (**D**–**F**) The total swimming distance, average swimming speed, and average escape latency of the first four training days at PND90. (**G**) The morris water maze trajectory maps of two groups of rats at PND60 and PND90. (**H**–**J**) The distance travelled and time spent in the target quadrant, as well as the number of crossings between the control group and the sevoflurane exposure group at PND60 and PND90. Cont: control group; Sevo: sevoflurane exposure group.

To evaluate the long-term neurofunctional impacts of early life repeated sevoflurane exposures, we conducted water-maze tests on PND60 and PND90. Despite clear alterations in brain metabolism and signs of neurotoxicity early after the sevoflurane exposures, our findings revealed no significant long-term memory impairments in the rats, as illustrated in Fig. 6. Additionally, there were no differences observed between sexes (Supplementary Fig. 12).

## Discussion

In this study, we have established the integrated use of metabolomic analysis and total RNA sequencing to investigate the impact of sevoflurane on the immature brain. Our study extends beyond immediate impacts, delving into the long-term consequences of this anesthetic. This holistic approach provides an in-depth understanding of the molecular and metabolic shifts caused by sevoflurane exposure, which is crucial for formulating safer anesthetic strategies in pediatric care.

Sevoflurane is commonly used in pediatric surgeries to correct various congenital abnormalities. Particularly in newborns, sevoflurane is essential for managing emergency procedures for complex heart defects, which may require multiple surgeries. The potential neurotoxic impact of sevoflurane, especially with repeated exposure during critical phases of brain development, is an area of ongoing research^3,21,22^.

In our study, we examined rats on PND 7 following exposure to sevoflurane and found notable metabolic changes. These changes, especially those affecting mitochondrial energy metabolism, align with existing knowledge about sevoflurane’s influence on mitochondrial function^23^. Notably, variations in metabolites like 3-methylglutarylcarnitine and butyrylcarnitine, essential for fatty acid metabolism, indicate mitochondrial dysfunction, which was suggested as a key contributor to anesthesia-induced neurotoxicity^24^. Moreover, changes in metabolites related to the TCA cycle and amino acid metabolism point to increased oxidative stress, a known result of mitochondrial dysfunction and also an event linking to anesthesia-induced neurotoxicity^25^.

Complementing these metabolic findings, our transcriptomic analysis reveals an upregulation in oxidative phosphorylation and DNA repair pathways. The WGCNA identified crucial gene modules, particularly the steelblue and brown modules, providing insight into the molecular mechanisms of sevoflurane-induced neurotoxicity. These modules, strongly correlated with sevoflurane exposure, play significant roles in DNA repair and mitochondrial functions. Additionally, miRNAs, key regulators of gene expression during brain development, may be altered by sevoflurane^26^, disrupting neurodevelopmental processes like synaptogenesis and neuronal growth. These disruptions likely contribute to the mitochondrial dysfunction and oxidative stress observed, suggesting a molecular basis for sevoflurane’s neurotoxic effects. Further studies are needed to explore these mechanisms. Further in-depth studies are necessary to explore these mechanisms in more detail.

**Fig. 7:**
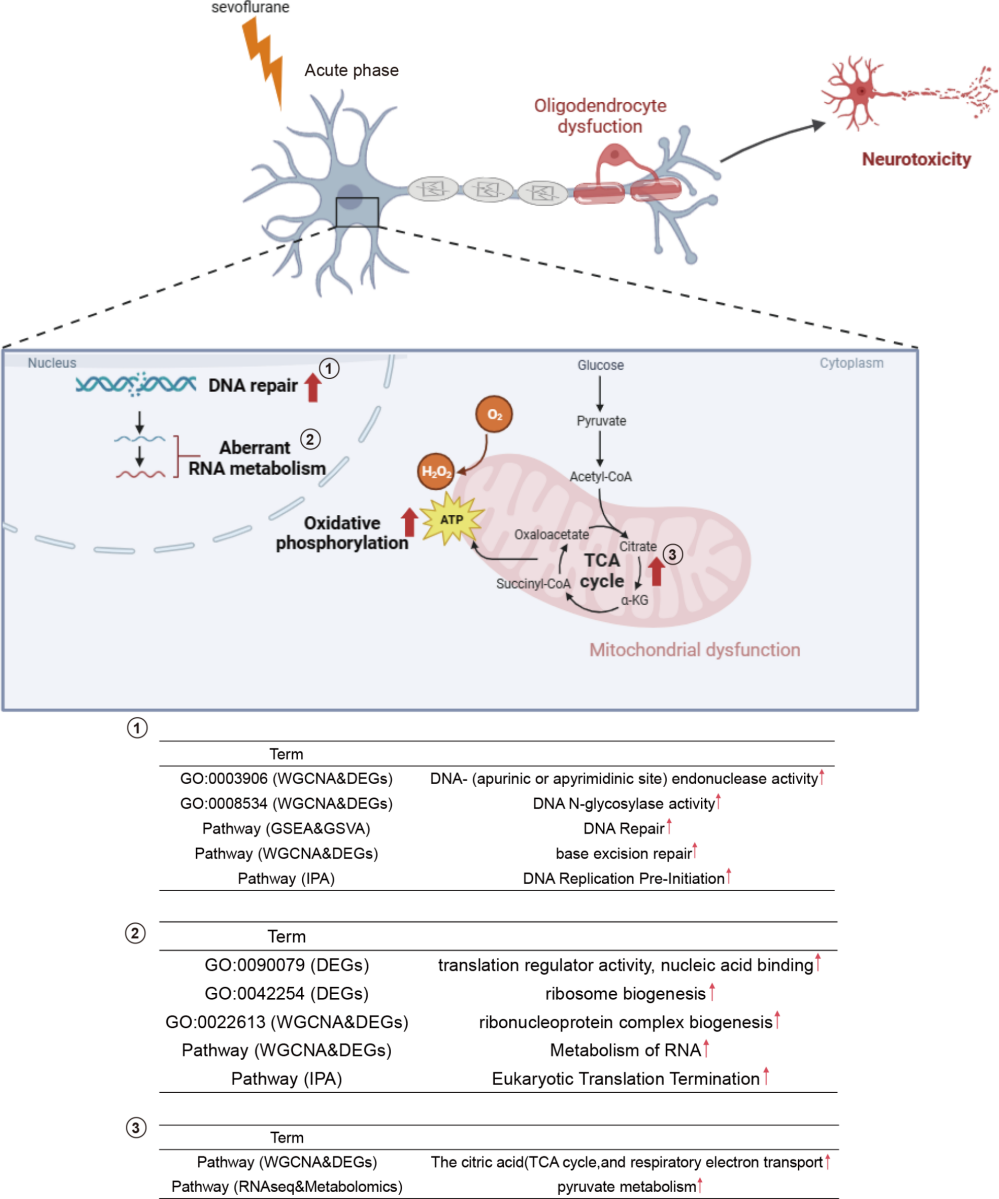
Cartoon illustration summarizing sevoflurane-induced effects. Exposure to sevoflurane induces abnormalities in DNA repair and RNA metabolism in the nuclei of rat hippocampal neurons, along with an increase in mitochondrial oxidative phosphorylation and TCA cycle activity, ultimately leading to neurodevelopmental impairment and neurotoxicity.

Neuronal cell death is a primary pathological outcome in response to sevoflurane exposure^11,12,27,28^, often coupled with intracellular oxidative stress. This stress disrupts cellular redox balance, amplifies TCA cycle activity, and augments mitochondrial oxidative phosphorylation, thus affecting mitochondrial functionality and energy metabolism. These disturbances are critical during the perinatal period, hindering the normal brain development and maturation in neonatal rat pups. Moreover, oxidative stress can directly impair DNA repair processes, leading to the accumulation of unrepaired DNA damage and reduced genomic stability. It also inflicts oxidative harm on RNA, disturbing vital processes like RNA synthesis, modification, and degradation, culminating in abnormal RNA metabolism. Both these impacts may impair the normal functioning and development of neuronal cells (Fig. 7).

The observed changes in gene expression, such as *Pax4* and *Gata3* genes, are crucial for neuronal development and functionality. Alterations in these genes could have long-term consequences on neurodevelopmental processes ^29 30^. Furthermore, pathway analyses indicating disturbances in mitochondrial function and synaptic specialization lend support to the notion that sevoflurane adversely affects neuronal growth and functionality. This is consistent with previous findings demonstrating the vulnerability of the developing brain to anesthetic agents^31^. The IPA analysis indicated potential cognitive effects, including motor dysfunction, epileptic disorders, and cognitive deficits. However, our behavioral tests showed no significant impacts. This discrepancy may stem from the difference in sensitivity between acute molecular responses from RNA-seq and long-term effects measured by behavioral tests. These findings highlight the need for further research on the long-term consequences of pediatric anesthesia, incorporating a wider range of behavioral assessments for a more comprehensive evaluation.

Our integrated analysis of metabolic and transcriptomic data has identified key pathways affected by sevoflurane, particularly those involved in pyruvate metabolism and amino acid biosynthesis, highlighting the need for a thorough approach to understand the impact of anesthesia on brain development. In rats treated with sevoflurane, we observed a significant increase in lactate and malate levels compared to controls, indicating again enhanced glycolysis and TCA cycle activity. These processes are crucial for fulfilling the high energy demands of the brain, where substances like glucose and malate are converted into pyruvate to participate in the TCA cycle, or they can be transformed into lactate through glycolysis.

Altogether, our findings indicate that sevoflurane exposure may amplify metabolic demands and oxidative stress in neuronal tissues, disrupting the energy balance and potentially impairing the overall health of neural cells. Such disruptions could lead to neurodevelopmental impairments. Therefore, our study contributes valuable insights into the field and underscores the need for ongoing research into the long-term effects of anesthetic exposure on early neurodevelopment.

Research into the potential neurotoxic mechanisms of sevoflurane has explored its impact on neural receptors, cell survival, stem cell growth, as well as the integrity of neural structures and barriers^11,12,32–35^. While our animal models showed short-term mitochondrial dysfunction and neurotoxicity from sevoflurane, these did not translate into significant long-term memory deficits, suggesting a possible resilience in the developing brain. We did not observe any sex differences in the water maze test following sevoflurane exposure, and our transcriptomic analysis showed similar changes in major biological processes and pathways across both sexes. While previous studies^36–39^, including Song et al. (2023)^40^, have reported sex-specific effects after neonatal sevoflurane exposure, this discrepancy may be due to differences in concentration, exposure duration, timing of assessments, and the specific tests used, as rodents have strong compensatory mechanisms. Additionally, earlier studies used single-nucleus RNA sequencing at PND 35, which is more sensitive to cell-type-specific effects, whereas our study employed bulk RNA sequencing at PND 9, a stage with less pronounced sex hormone influence. Further research is needed to fully understand potential sex-specific effects.

As a new generation inhaled anesthetic, sevoflurane is widely used in pediatric surgeries due to its favorable pharmacokinetic properties, including low blood and tissue solubility, short induction and recovery times, and no airway irritation^41^. It is particularly used in neonatal surgeries for complex conditions such as congenital esophageal atresia, diaphragmatic hernia, and various congenital heart defects, where prolonged or repeated exposure to anesthesia is often necessary.

Research on the neurodevelopmental impacts of repeated sevoflurane exposure during infancy has shown consistent evidence of neurotoxicity in animal models, but clinical findings are less definitive. Two major clinical trials, GAS^1^ and PANDA^2^, reported no significant cognitive impairments in children after single sevoflurane exposures. However, these studies were limited to single exposures. The 2018 MASK study^42^, which examined multiple exposures, also found no significant differences in intelligence but reported subtle deficits in processing speed, fine motor coordination, and reading abilities.

While sevoflurane has demonstrated neurotoxic effects and learning deficits in animal studies, these findings have not been conclusively replicated in humans. However, the neonatal rat model remains relevant for studying sevoflurane’s effects on neurodevelopment, as developmental processes are comparable between neonatal rats and human infants. Specifically, the brain growth spurt peaks around birth in humans and at PND7 in rodents^43,44^, making this model clinically relevant and suitable for exploring potential risks associated with prolonged or repeated sevoflurane exposure.

Our data contribute to this body of research by providing mechanistic insights into the potential neurotoxic effects of sevoflurane. Transcriptomic and metabolomic analyses revealed disruptions in mitochondrial function, oxidative phosphorylation, and neurodevelopmental pathways, suggesting early molecular and metabolic alterations. Even without evident cognitive deficits in clinical settings, underlying molecular changes could still occur, warranting further investigation.

Our study’s limitation lies in its focus solely on memory tests, which may not fully reflect the broad range of cognitive and neurological impacts of sevoflurane. Additionally, the possibility of delayed neurological effects emerging post-exposure highlights the need for more diverse cognitive assessments and extended monitoring to better understand sevoflurane’s effects, particularly in children.

In conclusion, while no significant long-term memory deficits were observed, our study reveals early molecular and metabolic disruptions from repeated sevoflurane exposure. Integrating metabolomic and transcriptomic data, we offer deeper insights into sevoflurane’s effects, laying the groundwork for future improvements in pediatric anesthetic practices and safer techniques for children.

## Electronic supplementary material

Below is the link to the electronic supplementary material.

Supplementary Material 1

Supplementary Material 2

Supplementary Material 3

Supplementary Material 4

Supplementary Material 5

Supplementary Material 6

Supplementary Material 7

Supplementary Material 8

Supplementary Material 9

Supplementary Material 10

Supplementary Material 11

Supplementary Material 12

Supplementary Material 13

Supplementary Material 14

Supplementary Material 15

Supplementary Material 16

Supplementary Material 17

Supplementary Material 18

Supplementary Material 19

Supplementary Material 20

Supplementary Material 21

Supplementary Material 22

Supplementary Material 23

Supplementary Material 24

Supplementary Material 25

Supplementary Material 26

## Data Availability

Data is provided within the manuscript or supplementary information files.
