# Supplementary material for: Sevoflurane exposure in early life: mitochondrial dysfunction and neurotoxicity in immature rat brains without long-term memory loss
Source: Sci Rep. 2024 Nov 20;14:28747. doi: 10.1038/s41598-024-79150-3 (PMC11579499; doi:10.1038/s41598-024-79150-3)
Supplement: Supplementary file 1 — Supplementary Material 1 [file 41598_2024_79150_MOESM1_ESM.pdf]

Sevoflurane exposure in early life: mitochondrial dysfunction and neurotoxicity in immature rat brains without long-term memory loss

Running title: Sevoflurane effect on immature brains

Lin Qiu[1,2]*, Hongwei Li[3,4,5]*, Bingbing Li[3], Joakim Ek[6], Xiaoli Zhang[3], Yiwen Chen[3], Zehua Shao[7], Jie Zhang[3], Jiaqiang Zhang[2], Hongqi Lin[1,2], Changlian Zhu[3,8], Yiran Xu[3#], Xiaoyang Wang[3,6,9#]

[1]Department of Anesthesia, Henan Provincial People's Hospital, Department of Anesthesia of Central China Fuwai Hospital, Central China Fu Wai Hospital of Zhengzhou University, Zhengzhou, 450003, Henan, China

[2]Zhengzhou University People's Hospital, Henan Provincial People's Hospital, 450003, China

[3]Henan Key Laboratory of Child Brain Injury and Henan Pediatric Clinical Research Center, Institute of Neuroscience and Third Affiliated Hospital of Zhengzhou University, Zhengzhou 450052, China

[4]Department of Laboratory Medicine, Third Affiliated Hospital of Zhengzhou University, Zhengzhou 450052, China

[5]Zhengzhou Key Laboratory for In Vitro Diagnosis of Hypertensive Disorders of Pregnancy, Zhengzhou 450052, China

[6]Centre of Perinatal Medicine & Health, Department of Physiology, Institute of Neuroscience and Physiology, University of Gothenburg, Gothenburg 40530, Sweden

[7]Department of Surgery, Fuwai Hospital, National Center for Cardiovascular Diseases, Chinese Academy of Medical Sciences and Peking Union Medical College, Beijing, 100037, China

[8]Center for Brain Repair and Rehabilitation, Institute of Neuroscience and Physiology, University of Gothenburg, Gothenburg 40530, Sweden

[9] Centre of Perinatal Medicine & Health, Department of Obstetrics and Gynaecology, Institute of Clinical Sciences, University of Gothenburg, Gothenburg 41685, Sweden

*Co-first authors; [#] Co-senior authors

Corresponding authors:

Xiaoyang Wang, Professor, Centre of Perinatal Medicine & Health, Department of Obstetrics and Gynaecology, Institute of Clinical Sciences, Sahlgrenska Academy, University of Gothenburg, Gothenburg 41685, Sweden. Phone: 0046 31 7863260. Email: xiaoyang.wang@fysiologi.gu.se

Lin Qiu, Professor, Department of anesthesia, Henan Provincial People's Hospital, Department of anesthesia of Central China Fuwai Hospital, Central China Fuwai Hospital of Zhengzhou University, Zhengzhou, Henan, 450003, China. Email: qiulin2005@163.com

Yiran Xu, Associate professor, Henan Key Laboratory of Child Brain Injury and Henan Pediatric Clinical Research Center, Institute of Neuroscience and the Third

Affiliated Hospital of Zhengzhou University, Zhengzhou, China. Email:
yiran.xu@zzuneuro.cn

Supplementary Figure Legends:

Supplementary Figure 1: Flowchart illustrating the detailed use of 80 rats in the study. Abbreviations: Sevo, sevoflurane; Cont, control.

Supplementary Figure 2: The key metabolic alterations in the immature rat brain induced by sevoflurane. A-D: PCA and OPLS-DA model score plots for positive (A, C) and negative (B, D) ionization modes. A, B: PCA score plots with metabolites color-coded (red: up-regulated, blue: down-regulated, gray: non-significant). C, D: 200 permutation test results for OPLS-DA. E and F: Heatmap of top differential metabolites in hippocampal tissue, comparing sevoflurane-treated and control groups for positive (E) and negative (F) ionization modes. Symbols '(+)' and '(−)' indicate positive and negative ionization modes.

Supplementary Figure 3: Pathway and enrichment analysis of differential metabolites induced by sevoflurane. (A) The enrichment pathways and (B) The pathway analysis of each biomarker. Analyses were conducted using the MetaboAnalysis 5.0 platform and based on data from the KEGG database.

Supplementary Figure 4: RNA sequencing reveals sevoflurane alters the gene expression in immature rat brain. (A) Principal Component Analysis (PCA) illustrating the distinct expression patterns of differentially expressed genes (DEGs). (B) A Volcano plot highlighting both upregulated and downregulated genes post-sevoflurane exposure. (C) A heatmap detailing the top 18 DEGs, with 6 of them prominently linked to neuronal development and

function.

Supplementary Figure 5: The functional enrichment analysis of DEGs in rats of different sexes. (A) A Volcano plot highlighting both upregulated and downregulated genes in male rats post-sevoflurane exposure. (B) A Volcano plot highlighting both upregulated and downregulated genes in female rats post-sevoflurane exposure. (C) The GO and KEGG enrichment results of DEGs in male rats. (D) The GO and KEGG enrichment results of DEGs in female rats. N = 10 (5 males, 5 females)/group

Supplementary Figure 6: Analysis of DEGs via IPA. The top 25 diseases or functions that show a decrease (top section) and an increase (bottom section) in association DEGs following sevoflurane exposure, as analyzed through IPA.

Supplementary Figure 7: IPA-generated regulatory effects on nervous system development and functions.The regulatory effects predicted by IPA related to nervous system development and functions. It includes all differentially regulated genes with a significance of $p < 0.001$. The analysis predicts upstream regulators acting through these genes, leading to downstream biological functions. The color coding (orange for increased, blue for decreased) indicates the predicted effect on these biological functions.

Supplementary Figure 8: Sevoflurane-induced gene alterations. The various gene alterations triggered by sevoflurane exposure, affecting several key neuronal functions. This included neuronal cell survival and death (Apoptosis, Necrosis, and Mitochondrial Function), neuronal adaptation (Neurogenesis and Synaptic Plasticity), and neuronal cellular regulation and communication (Neuroendocrine System and Oligodendrocytes).

Supplementary Figure 9: Identification of sevoflurane exposure related-hub genes and their associated functional enrichment analysis. (A) Left: Venn diagram of hub genes from DEGs and steelblue module; Right: GO and KEGG enrichment results. (B) Top: Venn diagram of hub genes from DEGs and brown module; Bottom: Metascape enrichment analysis.

Supplementary Figure 10: Integrated RNAseq and metabolomic analysis: common pathways in differentially expressed genes and metabolites. The chart shows KEGG pathways co-enriched in both transcriptome (orange bars) and metabolome (blue bars), with dashed lines indicating various significance levels. Symbols '(+)' and '(−)' indicate positive and negative ionization modes.

Supplementary Figure 11: Illustrated network of pyruvate metabolism. The interactions among metabolites involved in pyruvate metabolism, specifically L-lactic acid and L-malic acid, DEGs, and their potential implications in disease prediction.

Supplementary Figure 12: The learning and memory abilities of sevoflurane-exposed rats were assessed using the Morris water maze test, revealing no significant differences between sexes. The tests conducted at two time points: the first at PND60 (1st time point) and the second at PND90 (2nd time point). Two-way repeated measures ANOVA was used for statistical analysis. (A-C) Total swimming distance, average swimming speed, and average escape latency during the first four training days at PND60 in both male and female rats. (D-F) Total swimming distance, average swimming speed, and average escape latency during the first four training days at PND90 in both male and female rats. (G) The morris water maze trajectory maps of two groups of rats at PND60 and PND90. (H-J) Comparison of travel

distance, time spent, and number of crossings in the target quadrant between the control group and the sevoflurane exposure group at PND60 and PND90 for different sexes. Cont: control group; Sevo: sevoflurane exposure group. N = 20 (10 males, 10 females)/ group.
