# Supplementary figures and images for: Sevoflurane exposure in early life: mitochondrial dysfunction and neurotoxicity in immature rat brains without long-term memory loss
Source: Sci Rep. 2024 Nov 20;14:28747. doi: 10.1038/s41598-024-79150-3 (PMC11579499; doi:10.1038/s41598-024-79150-3)

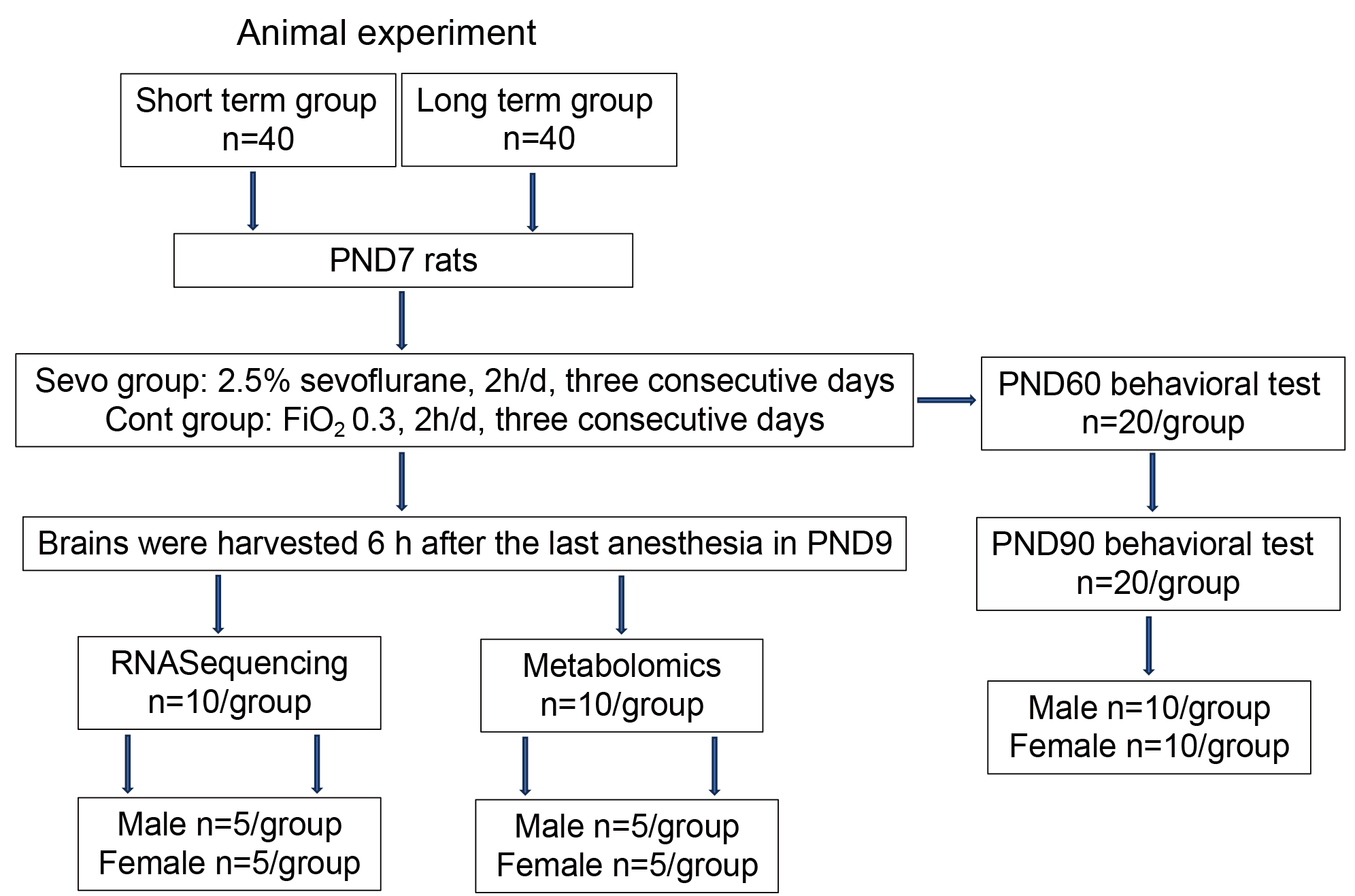

Supplement: Supplementary file 2 — Supplementary Material 2 [file 41598_2024_79150_MOESM2_ESM.tif]

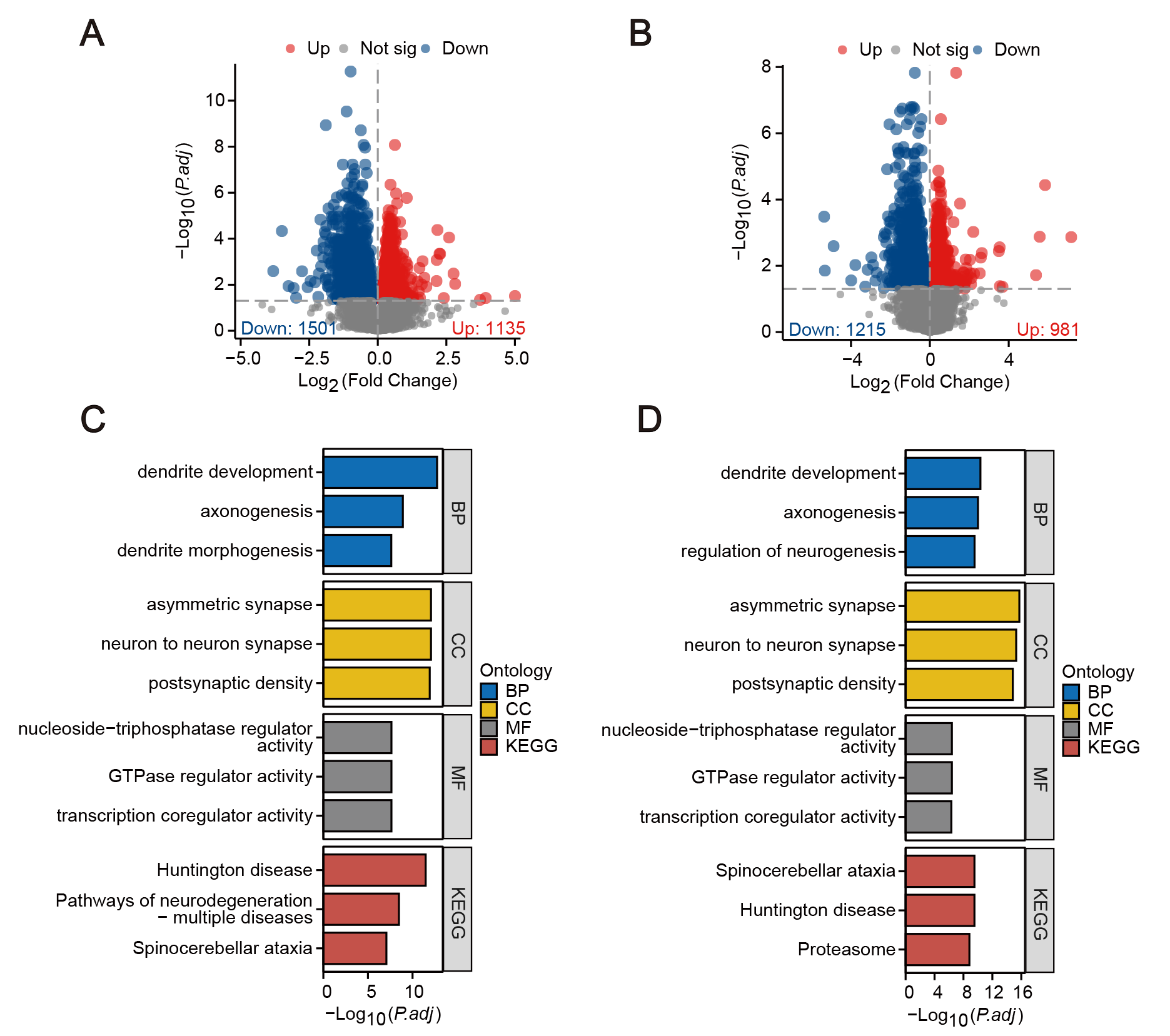

Supplement: Supplementary file 3 — Supplementary Material 3 [file 41598_2024_79150_MOESM3_ESM.tif]

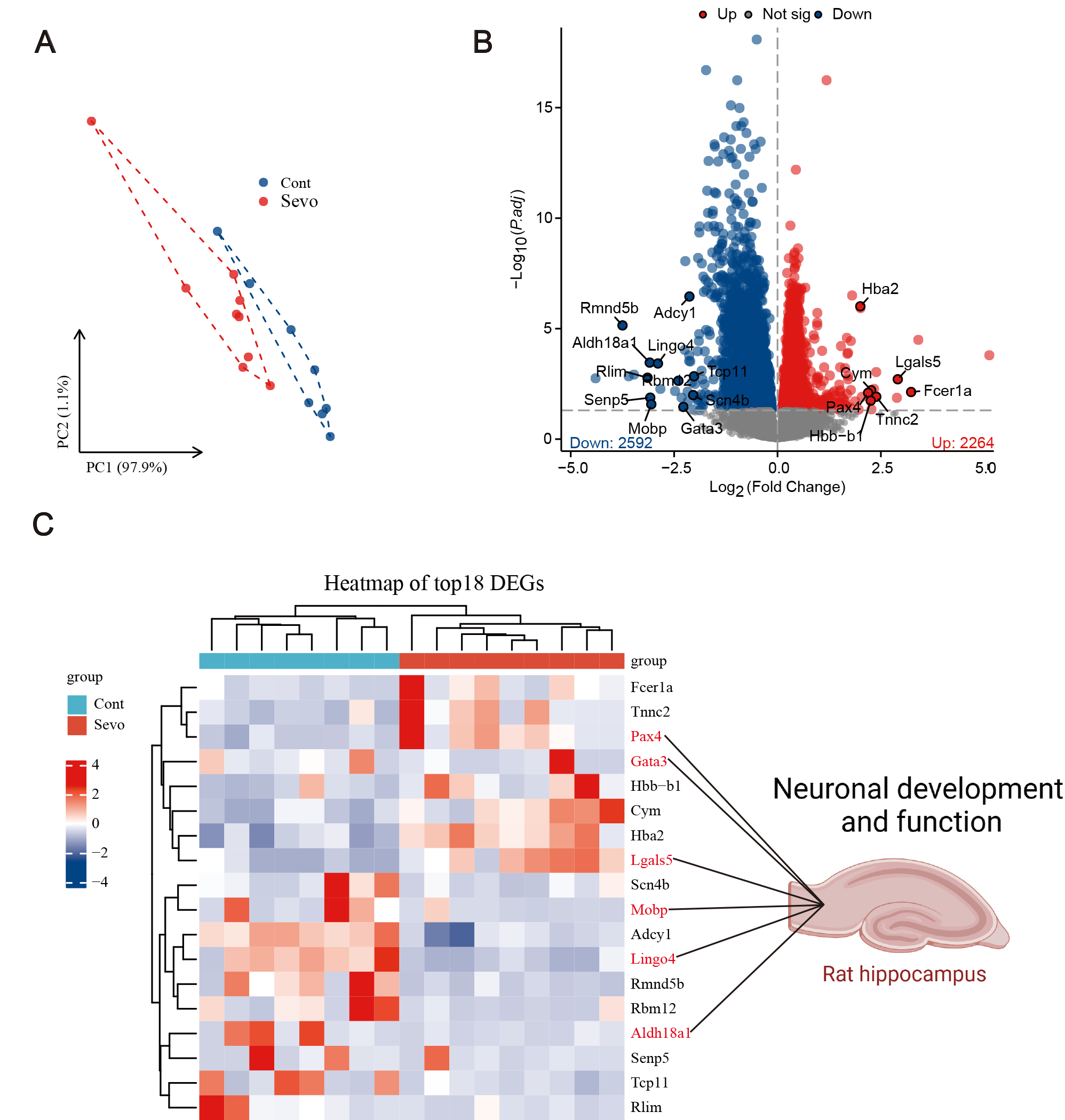

Supplement: Supplementary file 4 — Supplementary Material 4 [file 41598_2024_79150_MOESM4_ESM.tif]

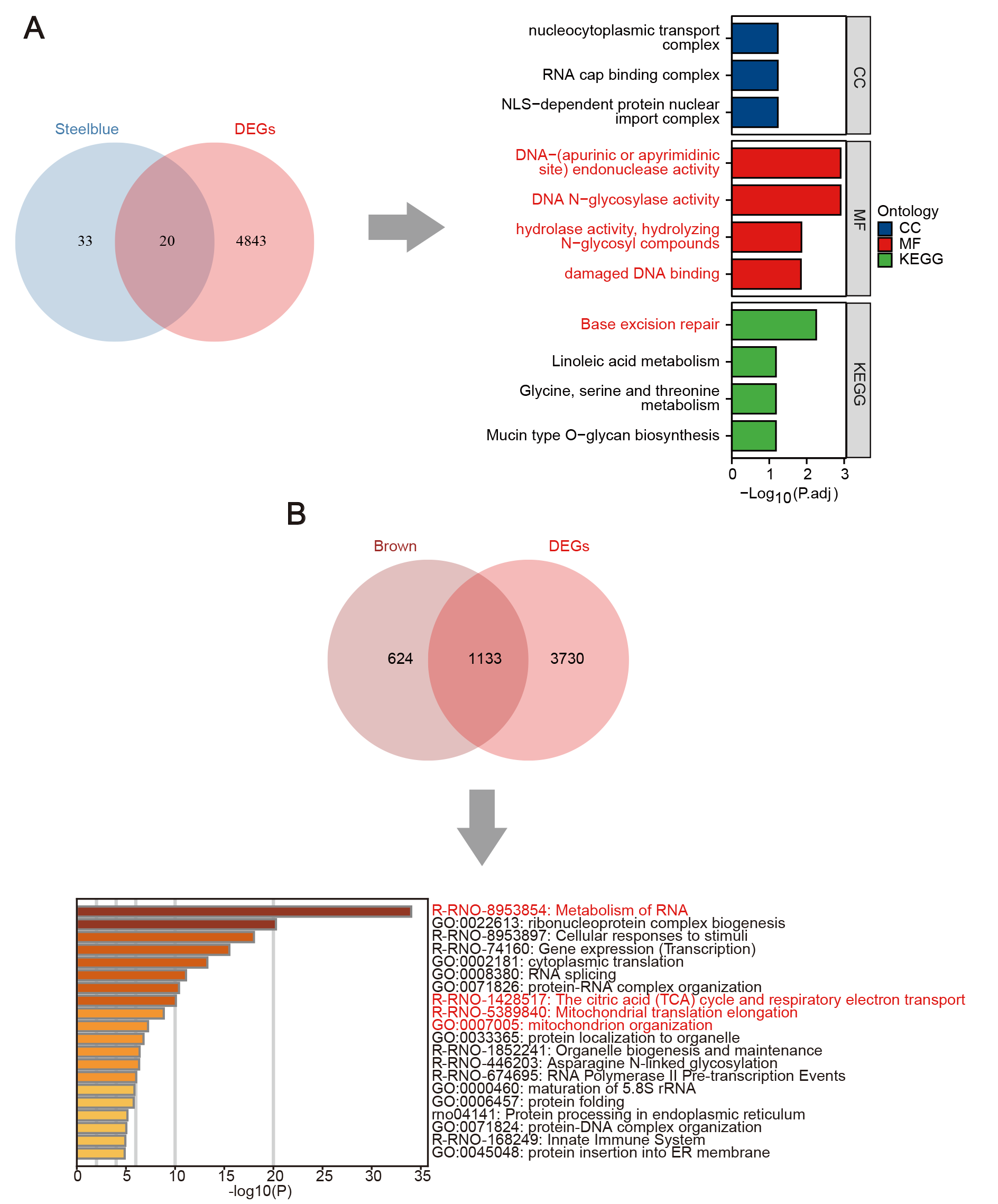

Supplement: Supplementary file 5 — Supplementary Material 5 [file 41598_2024_79150_MOESM5_ESM.tif]

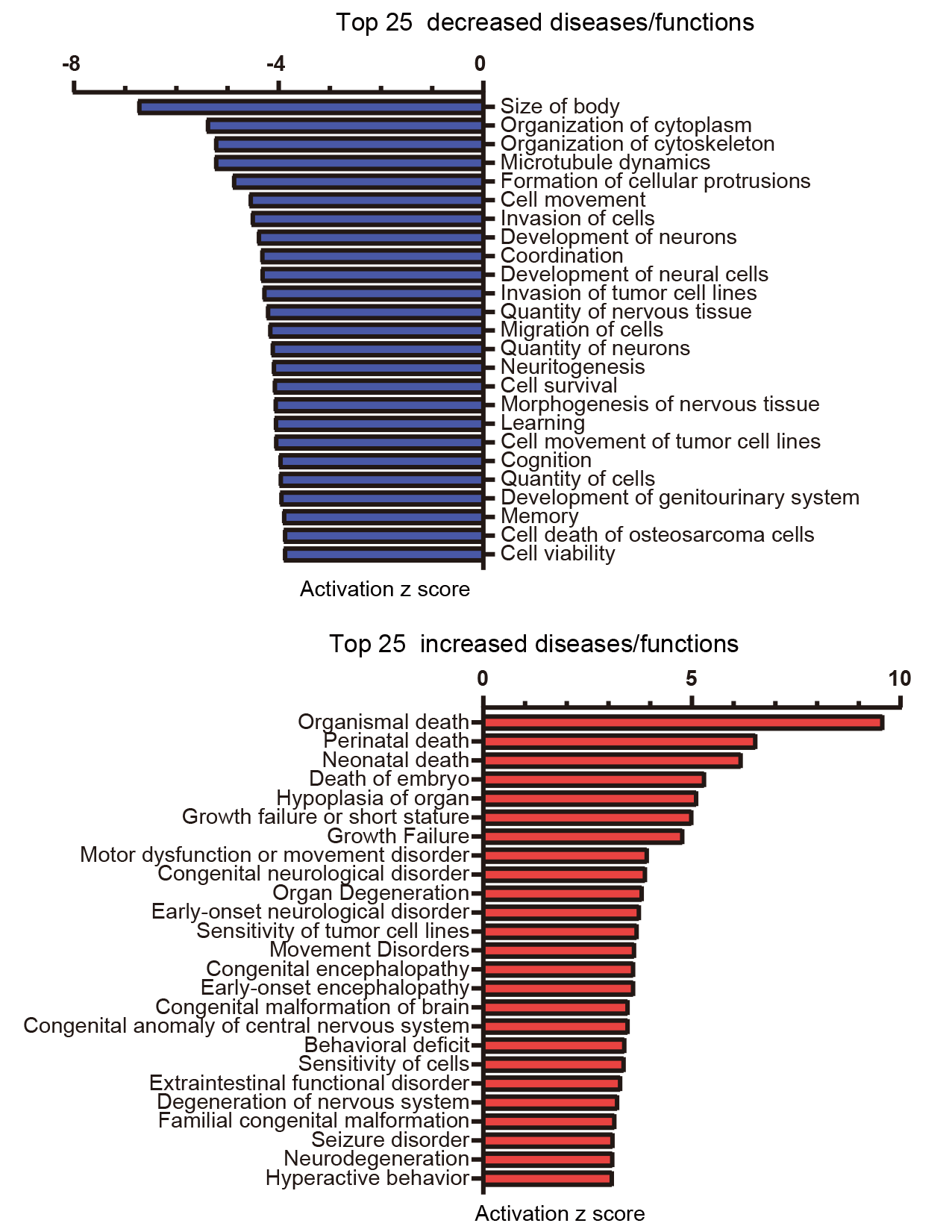

Supplement: Supplementary file 6 — Supplementary Material 6 [file 41598_2024_79150_MOESM6_ESM.tif]

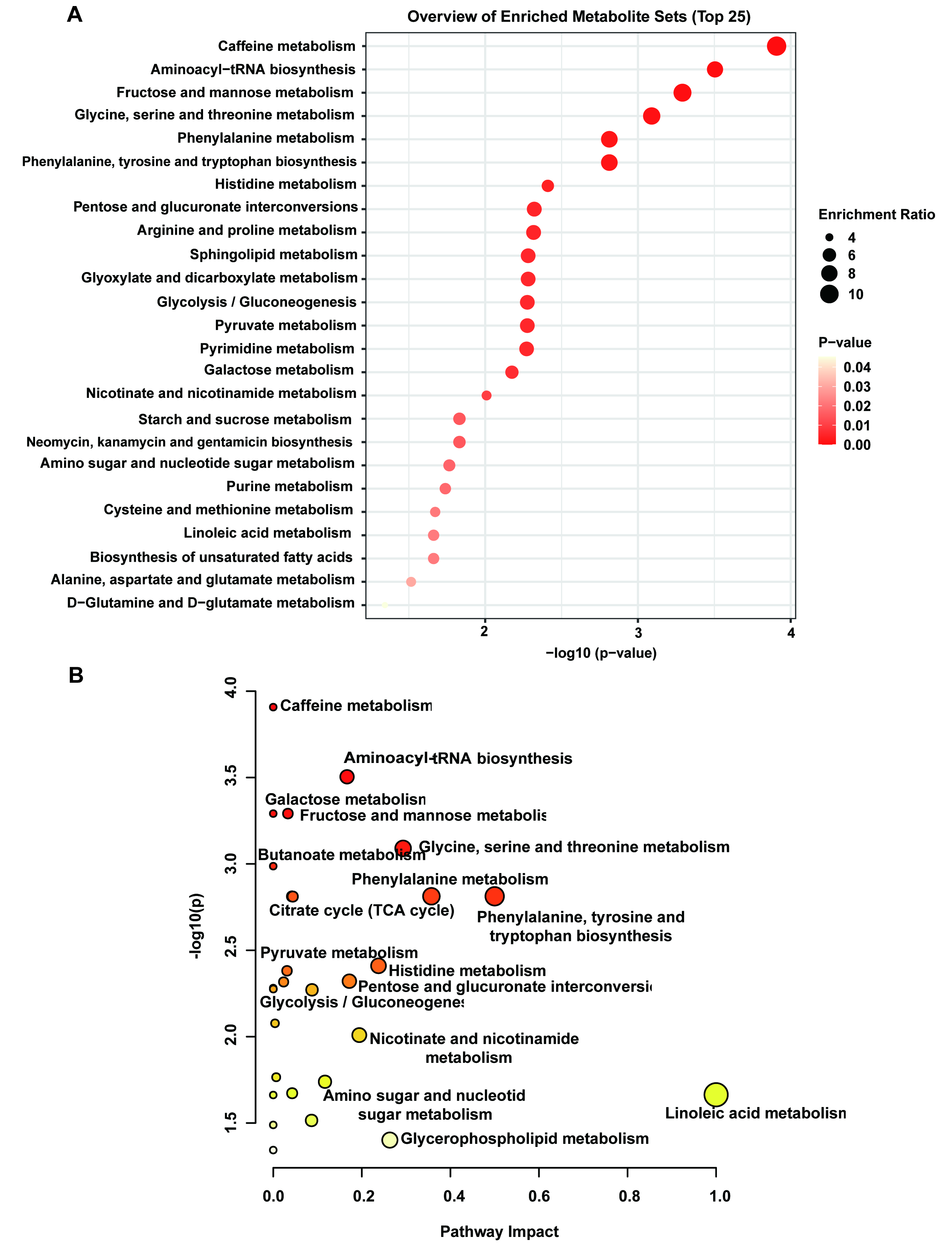

Supplement: Supplementary file 7 — Supplementary Material 7 [file 41598_2024_79150_MOESM7_ESM.tif]

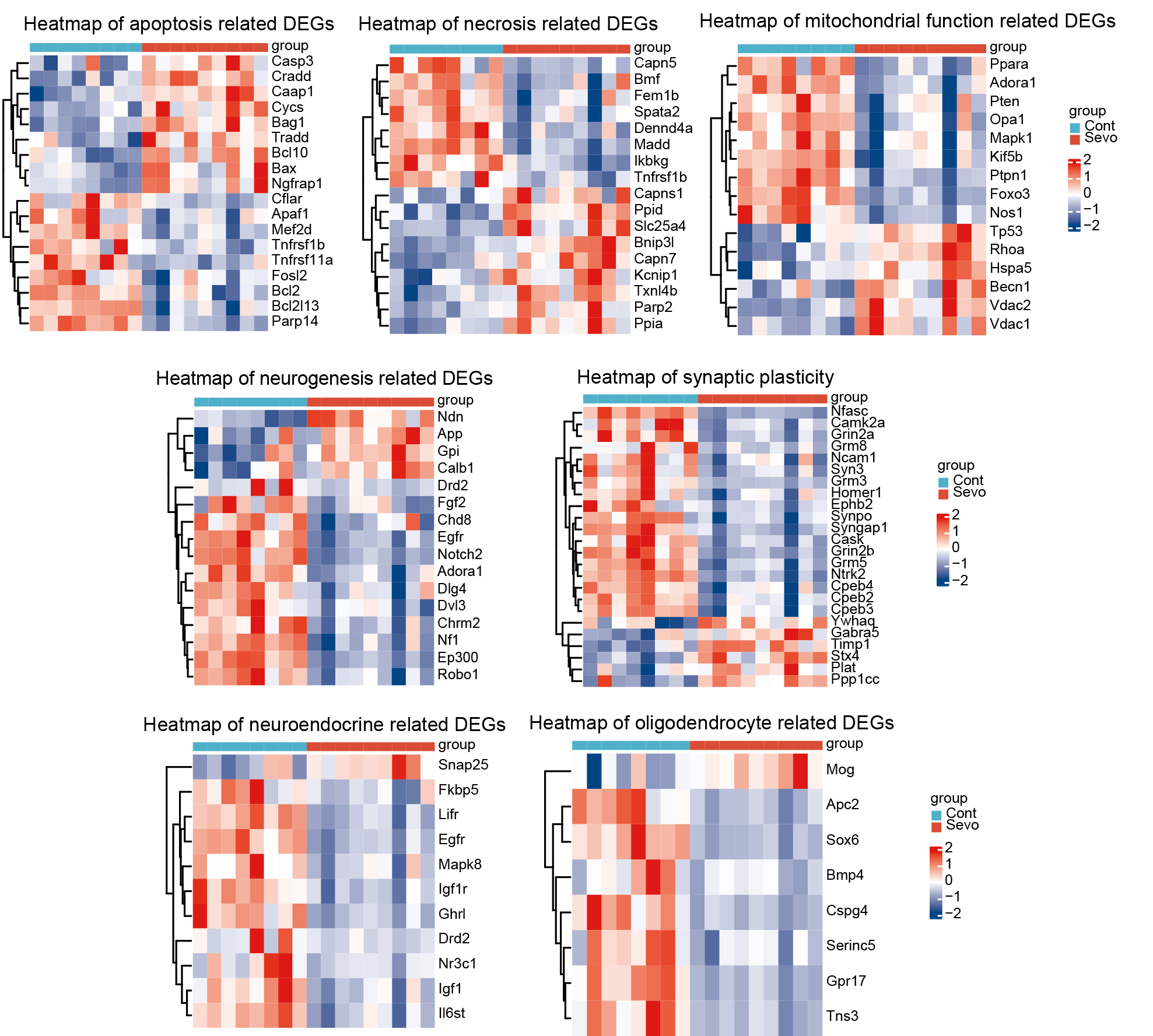

Supplement: Supplementary file 8 — Supplementary Material 8 [file 41598_2024_79150_MOESM8_ESM.tif]

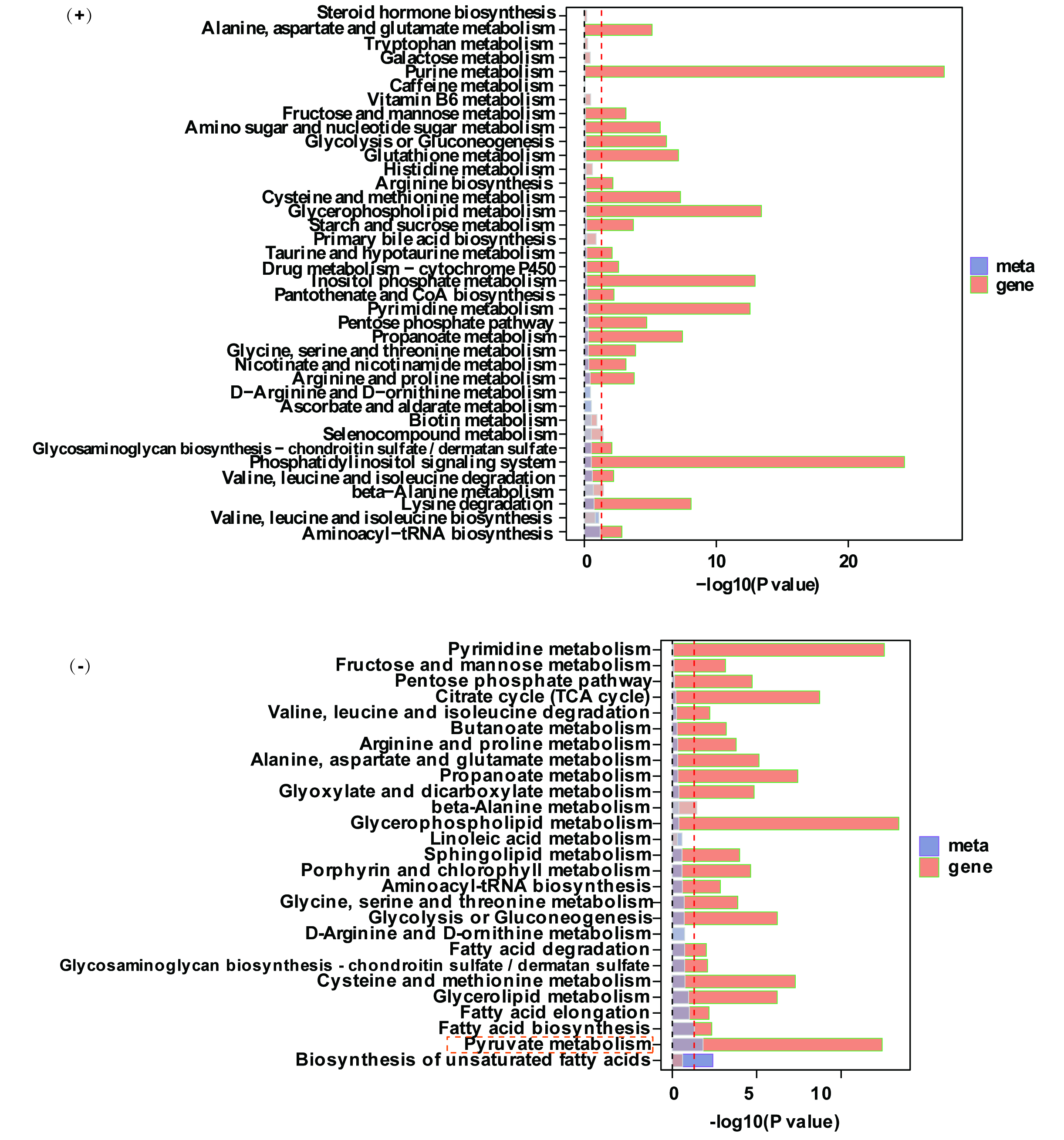

Supplement: Supplementary file 9 — Supplementary Material 9 [file 41598_2024_79150_MOESM9_ESM.tif]

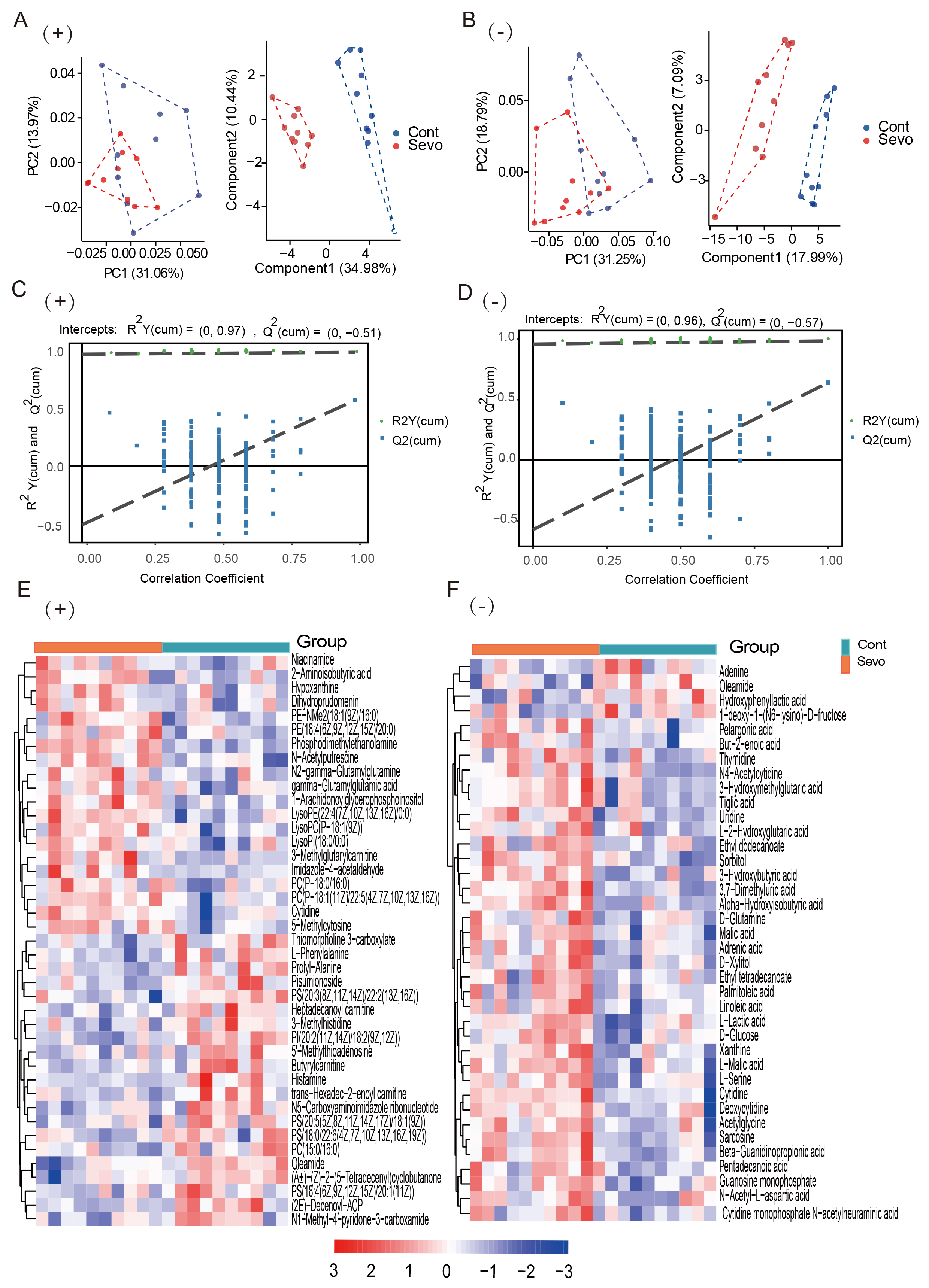

Supplement: Supplementary file 10 — Supplementary Material 10 [file 41598_2024_79150_MOESM10_ESM.tif]

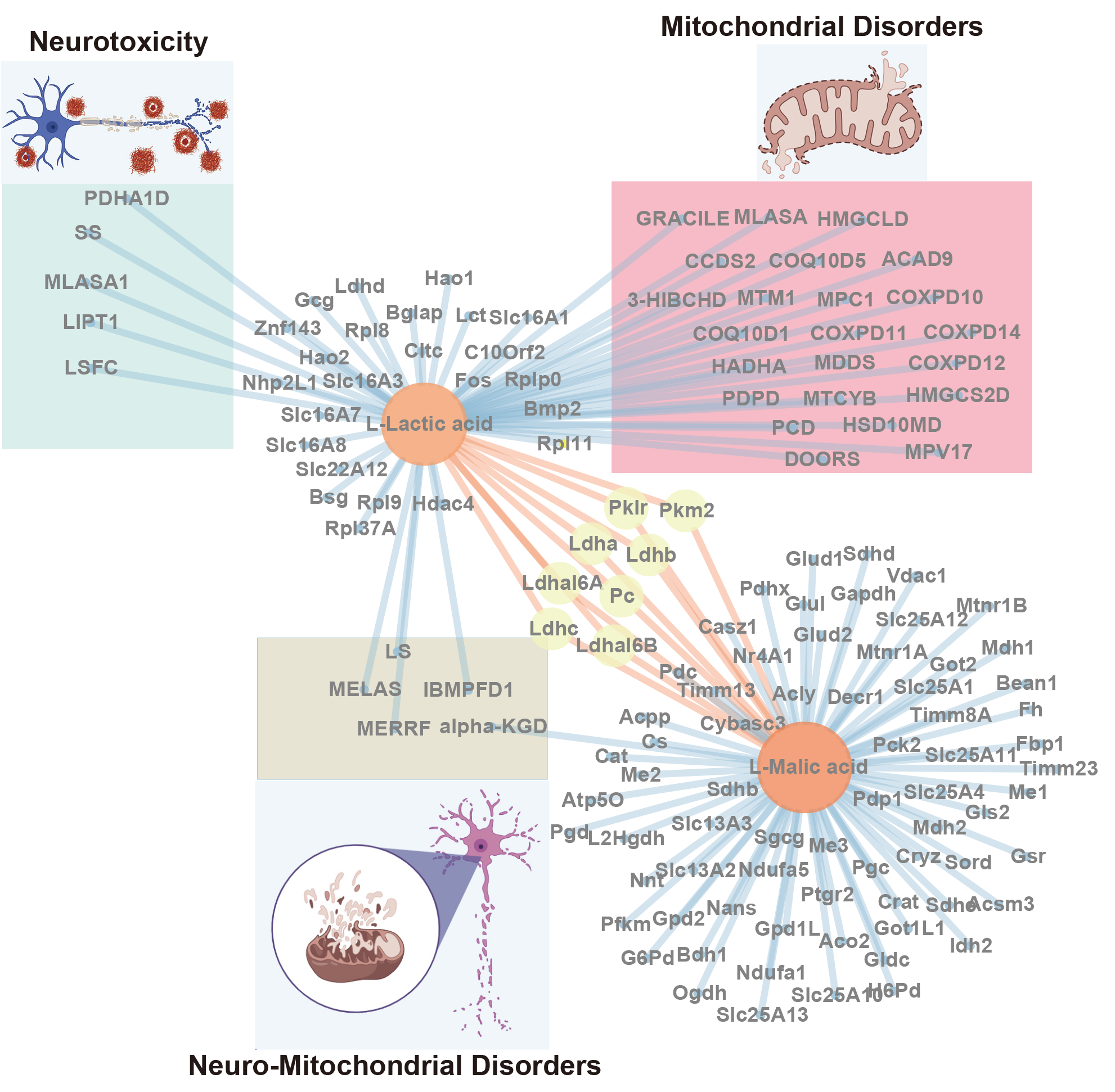

Supplement: Supplementary file 11 — Supplementary Material 11 [file 41598_2024_79150_MOESM11_ESM.tif]

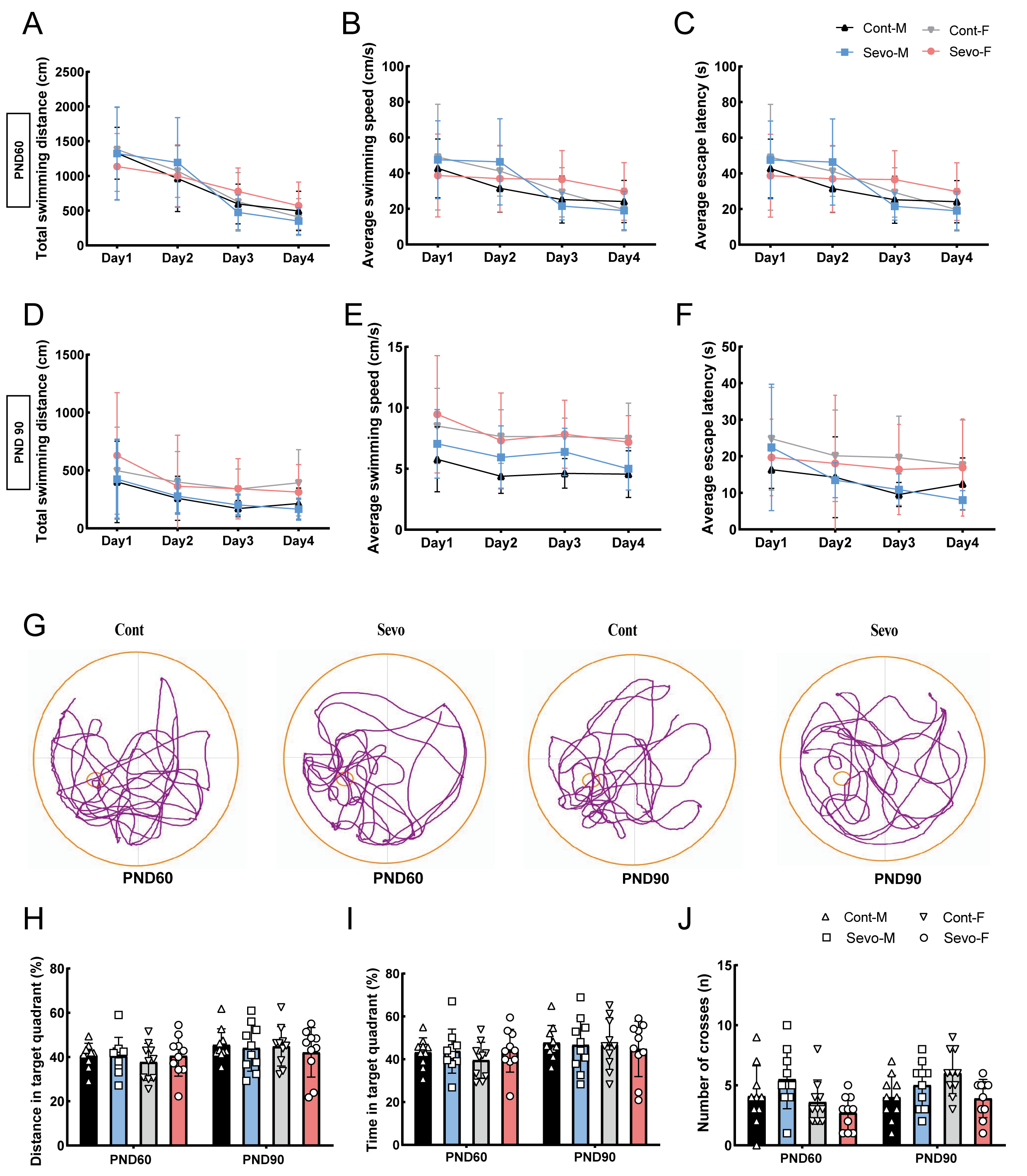

Supplement: Supplementary file 12 — Supplementary Material 12 [file 41598_2024_79150_MOESM12_ESM.tif]

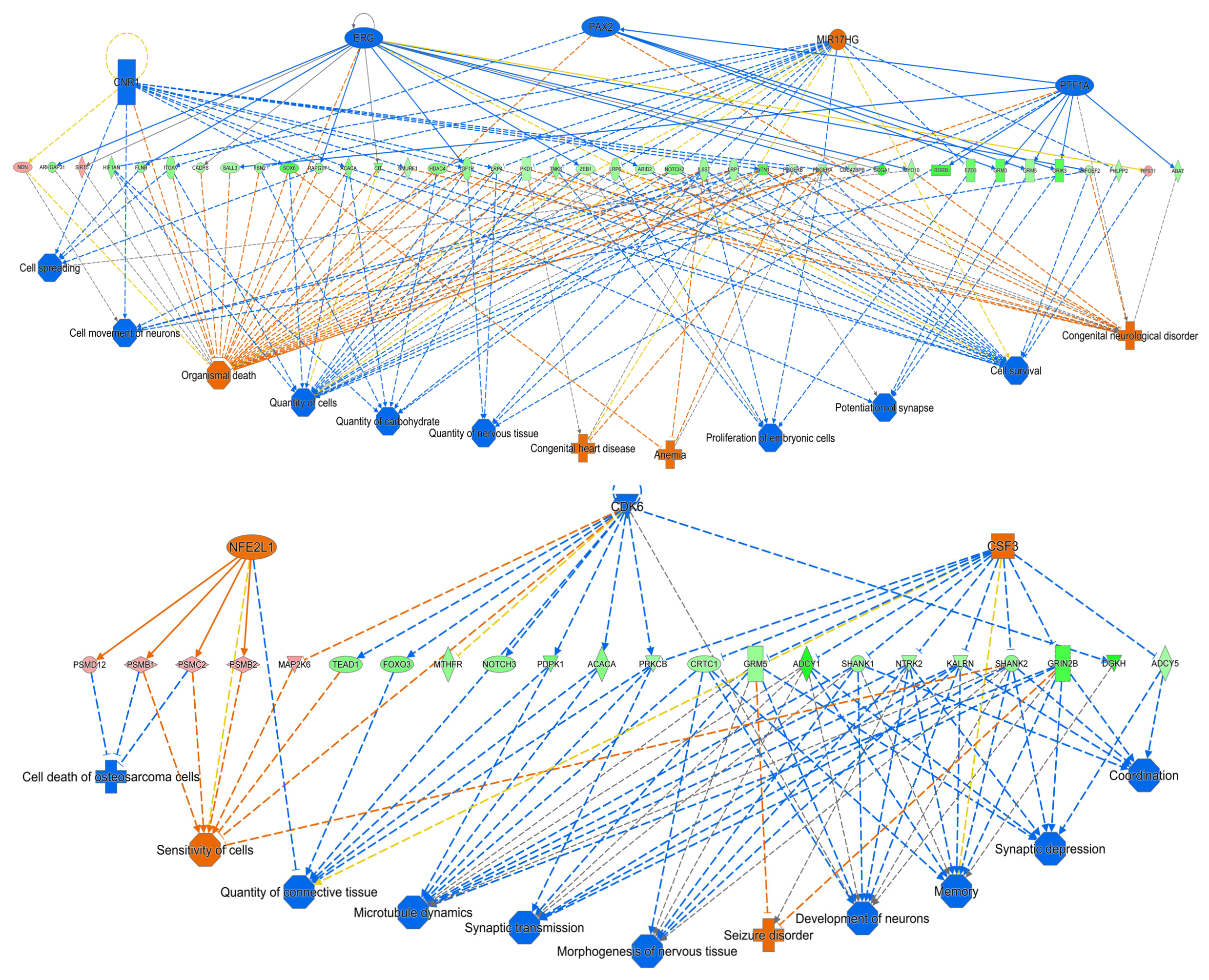

Supplement: Supplementary file 13 — Supplementary Material 13 [file 41598_2024_79150_MOESM13_ESM.tif]
