# Supplementary material for: Sevoflurane exposure in early life: mitochondrial dysfunction and neurotoxicity in immature rat brains without long-term memory loss
Source: Sci Rep. 2024 Nov 20;14:28747. doi: 10.1038/s41598-024-79150-3 (PMC11579499; doi:10.1038/s41598-024-79150-3)
Supplement: Supplementary file 24 — Supplementary Material 24 [file 41598_2024_79150_MOESM24_ESM.pdf]

Supplementary Table S1. The putative changed metabolites in rat hippocampal tissue induced by sevoflurane exposure and ranked by VIP value.

No	Sample ID	rt	mz	Super Class	VIP	P value	Q value	Fold change	Log fold change
1	Phosphodimethylethanolamine	464.08	170.06	Organic acids and derivatives	2.88	0.00	0.00	1.28	0.35
2	PI(20:2(11Z,14Z)/18:2(9Z,12Z))	220.17	887.56	Lipids and lipid-like molecules	2.56	0.00	0.04	0.88	-0.18
3	1-Arachidonoylglycerophosphoinositol	274.06	621.30	Lipids and lipid-like molecules	2.54	0.00	0.06	1.52	0.60
4	LysoPE(22:4(7Z,10Z,13Z,16Z)/0:0)	223.66	530.32	Lipids and lipid-like molecules	2.47	0.00	0.06	1.26	0.33
5	Alpha-Hydroxyisobutyric acid	293.43	103.04	Organic acids and derivatives	2.45	0.00	0.02	1.45	0.53
6	3,7-Dimethyluric acid	375.23	195.05	Organoheterocyclic compounds	2.43	0.00	0.02	1.62	0.69
7	L-Phenylalanine	283.22	166.09	Organic acids and derivatives	2.34	0.00	0.09	0.77	-0.37
8	Histamine	384.70	112.09	Organic nitrogen compounds	2.33	0.02	0.24	0.31	-1.69
9	Beta-Guanidinopropionic acid	369.38	130.06	Organic nitrogen compounds	2.23	0.00	0.03	1.11	0.15
10	3-Hydroxybutyric acid	252.91	103.04	Organic acids and derivatives	2.23	0.00	0.05	1.67	0.74

11	(2E)-Decenoyl-ACP	301.22	130.09	Organic acids and derivatives	2.22	0.01	0.19	0.64	-0.64
12	Sorbitol	174.72	181.07	Organic oxygen compounds	2.17	0.00	0.03	1.17	0.23
13	Sarcosine	369.24	88.04	Organic acids and derivatives	2.14	0.00	0.04	1.11	0.15
14	Pisumionoside	422.03	405.21	Lipids and lipid-like molecules	2.13	0.02	0.23	0.66	-0.60
15	3-Methylglutarylcarnitine	419.55	290.16	Lipids and lipid-like molecules	2.13	0.01	0.22	2.12	1.09
16	PE-NMe2(18:1(9Z)/16:0)	93.49	746.57	Lipids and lipid-like molecules	2.02	0.00	0.15	1.21	0.27
17	L-Malic acid	430.70	133.01	Organic acids and derivatives	2.00	0.00	0.05	1.38	0.46
18	Xanthine	237.63	151.03	Organoheterocyclic compounds	1.99	0.00	0.07	1.25	0.32
19	N4-Acetylcytidine	181.06	284.09	Nucleosides, nucleotides, and analogues	1.99	0.01	0.08	1.34	0.42
20	Prolyl-Alanine	357.08	187.11	Organic acids and derivatives	1.99	0.02	0.23	0.68	-0.55
21	N-Acetylputrescine	341.58	131.12	Organic acids and derivatives	1.96	0.00	0.16	1.15	0.20
22	PE(18:4(6Z,9Z,12Z,15Z)/20:0)	55.29	768.55	Lipids and lipid-like molecules	1.96	0.01	0.20	1.22	0.29

23	Thiomorpholine 3-carboxylate	349.62	148.04	Organic acids and derivatives	1.95	0.01	0.22	0.86	-0.22
24	Uridine	175.65	243.06	Nucleosides, nucleotides, and analogues	1.95	0.01	0.08	1.15	0.21
25	L-Lactic acid	243.06	89.02	Organic acids and derivatives	1.94	0.01	0.08	1.15	0.20
26	L-Serine	397.71	104.03	Organic acids and derivatives	1.88	0.01	0.08	1.18	0.24
27	D-Xylitol	98.05	151.06	Organic oxygen compounds	1.88	0.00	0.07	1.22	0.28
28	Oleamide	33.84	282.28	Lipids and lipid-like molecules	1.87	0.01	0.18	0.82	-0.29
29	Tiglic acid	397.71	99.04	Lipids and lipid-like molecules	1.86	0.01	0.11	1.16	0.22
30	3-Methylhistidine	428.12	170.09	Organic acids and derivatives	1.85	0.02	0.24	0.78	-0.35
31	3-Hydroxymethylglutaric acid	397.707	161.0451	Lipids and lipid-like molecules	1.85	0.02	0.12	1.15	0.20
32	Imidazole-4-acetaldehyde	124.799	111.0554	Organoheterocyclic compounds	1.82	0.04	0.27	1.92	0.94
33	Adrenic acid	37.7136	331.2644	Lipids and lipid-like molecules	1.80	0.01	0.09	1.29	0.37
34	PC(P-18:1(11Z)/22:5(4Z,7Z,10Z,13Z,16Z))	163.958	818.6037	Lipids and lipid-like molecules	1.77	0.01	0.17	1.26	0.34

35	2-Aminoisobutyric acid	322.162	104.0708	Organic acids and derivatives	1.76	0.01	0.22	1.26	0.33
36	Malic acid	588.3575	133.0138	Organic acids and derivatives	1.73	0.01	0.08	1.18	0.24
37	Niacinamide	59.703	123.0551	Organoheterocyclic compounds	1.72	0.03	0.27	1.06	0.09
38	gamma-Glutamylglutamic acid	488.9695	277.1028	Organic acids and derivatives	1.71	0.03	0.25	1.19	0.25
39	Cytidine	262.377	244.0923	Nucleosides, nucleotides, and analogues	1.70	0.01	0.20	1.24	0.31
40	N2-gamma-Glutamylglutamine	456.0805	276.1184	Organic acids and derivatives	1.69	0.03	0.26	1.20	0.26
41	D-Glucose	243.048	179.0557	Organic oxygen compounds	1.69	0.01	0.11	1.15	0.20
42	Acetylglycine	336.542	116.0346	Organic acids and derivatives	1.68	0.01	0.11	1.14	0.18
43	5-Methylcytosine	220.117	126.0662	Organoheterocyclic compounds	1.68	0.02	0.24	1.24	0.31
44	Thymidine	93.8949	241.0827	Nucleosides, nucleotides, and analogues	1.67	0.02	0.13	1.20	0.27
45	Hypoxanthine	471.593	137.0455	Organoheterocyclic compounds	1.67	0.04	0.28	1.17	0.23
46	LysoPC(P-18:1(9Z))	213.469	506.3611	Lipids and lipid-like molecules	1.67	0.02	0.24	1.29	0.37

47	But-2-enoic acid	96.1499	85.02883	Lipids and lipid-like molecules	1.67	0.01	0.09	1.20	0.26
48	Guanosine monophosphate	487.382	362.0511	Nucleosides, nucleotides, and analogues	1.67	0.02	0.13	1.16	0.22
49	Dihydroprudomenin	487.166	495.148	Phenylpropanoids and polyketides	1.65	0.05	0.30	1.27	0.34
50	Heptadecanoyl carnitine	203.355	414.358	Lipids and lipid-like molecules	1.65	0.03	0.26	0.76	-0.40
51	PC(15:0/16:0)	162.152	720.5531	Lipids and lipid-like molecules	1.64	0.03	0.26	0.84	-0.25
52	LysoPI (18:0/0:0)	275.61	601.3359	Lipids and lipid-like molecules	1.64	0.04	0.29	1.17	0.22
53	trans-Hexadec-2-enoyl carnitine	206.456	398.3268	Lipids and lipid-like molecules	1.64	0.04	0.29	0.82	-0.29
54	Butyrylcarnitine	282.329	232.1542	Lipids and lipid-like molecules	1.64	0.02	0.25	0.60	-0.74
55	Adenine	250.162	134.0467	Organoheterocyclic compounds	1.62	0.02	0.14	0.77	-0.37
56	5'-Methylthioadenosine	92.899	298.0957	Nucleosides, nucleotides, and analogues	1.62	0.04	0.27	0.93	-0.11
57	Cytidine monophosphate N-acetylneuraminic acid	467.382	613.1417	Nucleosides, nucleotides, and analogues	1.59	0.02	0.12	1.19	0.25

58	PS(20:3(8Z,11Z,14Z)/22:2(13Z,16Z))	219.299	866.5914	Lipids and lipid-like molecules	1.57	0.02	0.24	0.75	-0.42
59	PC(P-18:0/16:0)	155.789	746.6052	Lipids and lipid-like molecules	1.57	0.05	0.29	1.16	0.22
60	PS(18:4(6Z,9Z,12Z,15Z)/20:1(11Z))	221.013	810.5281	Lipids and lipid-like molecules	1.56	0.05	0.29	0.91	-0.13
61	N5-Carboxyaminoimidazole ribonucleotide	327.81	340.0554	Organic oxygen compounds	1.56	0.02	0.24	0.80	-0.32
62	Hydroxyphenyllactic acid	212.046	181.0505	Phenylpropanoids and polyketides	1.54	0.02	0.13	0.82	-0.29
63	L-2-Hydroxyglutaric acid	197.541	147.0295	Lipids and lipid-like molecules	1.54	0.04	0.18	1.11	0.15
64	Linoleic acid	37.2826	279.2335	Lipids and lipid-like molecules	1.53	0.02	0.13	1.18	0.24
65	1-deoxy-1-(N6-lysino)-D-fructose	486.6	132.0298	Organic acids and derivatives	1.51	0.05	0.18	0.85	-0.23
66	Pelargonic acid	52.6717	157.1229	Lipids and lipid-like molecules	1.50	0.02	0.13	1.17	0.23
67	N-Acetyl-L-aspartic acid	420.622	174.0404	Organic acids and derivatives	1.49	0.03	0.15	1.08	0.11
68	PS(20:5(5Z,8Z,11Z,14Z,17Z)/18:1(9Z))	221.898	808.5121	Lipids and lipid-like molecules	1.47	0.03	0.27	0.89	-0.16
69	PS(18:0/22:6(4Z,7Z,10Z,13Z,16Z,19Z))	220.172	836.5428	Lipids and lipid-like molecules	1.46	0.05	0.30	0.93	-0.11
70	N1-Methyl-4-pyridone-3-carboxamide	84.95355	153.0655	Organoheterocyclic compounds	1.44	0.03	0.26	0.57	-0.82

71	Deoxycytidine	227.422	226.0831	Nucleosides, nucleotides, and analogues	1.42	0.02	0.13	1.25	0.32
72	Ethyl dodecanoate	97.0443	227.2013	Lipids and lipid-like molecules	1.40	0.03	0.16	1.16	0.21
73	D-Glutamine	394.479	145.0611	Organic acids and derivatives	1.40	0.05	0.18	1.14	0.19
74	Ethyl tetradecanoate	96.752	255.2331	Lipids and lipid-like molecules	1.37	0.04	0.17	1.08	0.11
75	Pentadecanoic acid	44.1779	241.2174	Lipids and lipid-like molecules	1.35	0.04	0.17	1.23	0.30
76	Palmitoleic acid	37.2826	253.2171	Lipids and lipid-like molecules	1.33	0.04	0.18	1.20	0.26
77	(Z)-2-(5-Tetradecenyl)cyclobutanone	33.342	265.2521	Organic oxygen compounds	1.13	0.02	0.24	0.73	-0.45

Supplementary Table S2. The most markedly changed metabolites identified by VIP>1 and fold change >1.5 or <0.6 and related pathways or diseases.

No	Sample ID	rt	mz	Super Class	VIP	Fold change	Log fold change	Related pathways	Related diseases	Change trend compared with the control
1	3-Methylglutarylcarnitine	419.55	290.16	Lipids and lipid-like molecules	2.13	2.12	1.09	Leucine metabolism, mitochondrial energy metabolism	TBI, EA	Upregulated
2	3-Hydroxybutyric acid	252.91	103.04	Organic acids and derivatives	2.23	1.67	0.74	Butanoate metabolism	MDD, BD, Epilepsy, ASD	Upregulated
3	3,7-Dimethyluric acid	375.23	195.05	Organoheterocyclic compounds	2.43	1.62	0.69	Caffeine metabolism	Dementia, Cognitive function	Upregulated
4	1-Arachidonoylglycerophosphoinositol	274.06	621.31	Lipids and lipid-like molecules	2.54	1.52	0.60	Lipid metabolism	-	Upregulated
5	Butyrylcarnitine	282.33	232.15	Lipids and lipid-like molecules	1.64	0.60	-0.74	Fatty acid metabolism, Mitochondrial energy metabolism	HIE	Downregulated

6	N1-Methyl-4-pyridone-3-carboxamide	84.95	153.07	Organoheterocyclic compounds	1.44	0.57	-0.82	Nicotinate and nicotinamide metabolism	-	Downregulated
7	Histamine	384.70	112.09	Organic nitrogen compounds	2.33	0.31	-1.69	Histidine metabolism	AD, PD	Downregulated

TBI: traumatic brain injury; EA: emergence agitation; MDD: Major depressive disorder; BD: Bipolar disorder; ASD: Autism spectrum disorder; HIE: Hypoxic-ischemic encephalopathy; AD: Alzheimer's disease; PD: Parkinson’s disease.

Supplementary Table S3. Metabolites-genes overlapping pathway results for KEGG co-enrichment analysis.

Ion mode	KEGG pathway	Gene P-value	Metabolite P-value	Hits_gene	Hits_metabolites
positive	Aminoacyl-tRNA biosynthesis	0.001	0.063	9	8
positive	Valine, leucine and isoleucine biosynthesis	0.148	0.079	1	3
positive	Lysine degradation	0.184	< 0.001	15	4
positive	beta-Alanine metabolism	0.21	0.035	4	2
negative	Pyruvate metabolism	0.015	< 0.001	16	4
negative	Biosynthesis of unsaturated fatty acids	0.004	0.245	1	4
negative	Fatty acid biosynthesis	0.049	0.004	4	2

Abbreviations

3-HIBCHD	3-Hydroxyisobutyryl-CoA Hydrolase Deficiency
ACAD9	Mitochondrial Complex I Deficiency due to ACAD9 Deficiency
alpha-KGD	Alpha-Ketoglutarate Dehydrogenase Deficiency
CCDS2	Cerebral Creatine Deficiency Syndrome 2
COQ10D1	Coenzyme Q10 Deficiency, Primary, 1
COQ10D5	Coenzyme Q10 Deficiency, Primary, 5
COXPD10	Combined Oxidative Phosphorylation Deficiency 10
COXPD11	Combined Oxidative Phosphorylation Deficiency 11
COXPD12	Combined Oxidative Phosphorylation Deficiency 12
COXPD14	Combined Oxidative Phosphorylation Deficiency 14
DOORS	Deafness, Onychodystrophy, Osteodystrophy, Mental Retardation, and Seizures Syndrome
GRACILE	GRACILE Syndrome
HADHA	Mitochondrial Trifunctional Protein Deficiency
HMGCLD	3-Hydroxy-3-Methylglutaryl-CoA Lyase Deficiency
HMGCS2D	3-Hydroxy-3-Methylglutaryl-CoA Synthase-2 Deficiency
HSD10MD	HSD10 Mitochondrial Disease
IBMPFD1	Inclusion Body Myopathy with Early-Onset Paget Disease with or without Frontotemporal Dementia 1
LIPT1	Lipoyltransferase 1 Deficiency
LS	Leigh Syndrome
LSFC	Leigh Syndrome, French Canadian Type
MDDS	Mitochondrial DNA Depletion Syndrome 5 (Encephalomyopathic with or without Methylmalonic Aciduria)
MELAS	Mitochondrial Encephalomyopathy, Lactic Acidosis, and Stroke-Like Episodes
MERRF	Myoclonic Epilepsy Associated with Ragged-Red Fibers

MLASA	Myopathy with Lactic Acidosis, Hereditary
MLASA1	Myopathy, Lactic Acidosis, and Sideroblastic Anemia 1
MPC1	Mitochondrial Pyruvate Carrier Deficiency
MPV17	Mitochondrial Phosphate Carrier Deficiency
MTCYB	Mitochondrial Complex IV Deficiency
MTM1	Mitochondrial Myopathy, Infantile, Transient
PCD	Pyruvate Carboxylase Deficiency
PDHA1D	Pyruvate Dehydrogenase E1-Alpha Deficiency
PDPD	Pyruvate Dehydrogenase Phosphatase Deficiency
SS	Sengers Syndrome
